# *MTHFR* 677C/T gene polymorphism and dietary habits: effects on trace element levels, amino acids, and biochemical parameters

**DOI:** 10.3389/fnut.2025.1710613

**Published:** 2026-01-12

**Authors:** Tatjana Orct, Jelena Kovačić, Ines Peremin, Zorana Kljaković-Gašpić, Daria Pašalić, Ankica Sekovanić, Adrijana Dorotić, Blanka Tariba Lovaković, Andreja Jurič, Alica Pizent, Fran Crnjac, Lora Dukić, Marko Gerić, Ivone Jakaša, Goran Gajski

**Affiliations:** 1 Institute for Medical Research and Occupational Health, Zagreb, Croatia; 2 Faculty of Food Technology and Biotechnology, University of Zagreb, Zagreb, Croatia; 3 School of Medicine, University of Zagreb, Zagreb, Croatia; 4 University Hospital Sveti Duh, Zagreb, Croatia; 5 University Hospital Center Rijeka, Rijeka, Croatia

**Keywords:** single nucleotide polymorphism, recessive genotype, toxic and essential elements, folate, proline, glutamic acid

## Abstract

**Background:**

Previous research has identified that, in individuals with lower folate and/or vitamin B_12_ levels, homocysteine is associated with specific elements, while amino acids are associated with folate, thereby influencing folate availability during supplementation. This study investigates the impact of the *MTHFR* 677C/T polymorphism on levels of these compounds in individuals with differing meat consumption preferences, non-vegetarians and vegetarians.

**Methods:**

The study was conducted on 162 Croatian subjects. *MTHFR* gene polymorphism was determined by PCR-RFLP, elements by ICP-QQQ, amino acids by GC–MS, and biochemical parameters by chemiluminescent immunoassay and enzymatic methods. Differences between the groups were tested by ANOVA, while associations between the parameters were examined by multiple regression analyses.

**Results:**

The CT + TT genotype had lower folate levels in comparison to the CC genotype. The influence of *MTHFR* 677C/T polymorphism on element levels was limited to Al and Ca: individuals with the CT + TT genotype exhibited higher plasma Ca levels than the wild-type (CC) genotype, while lower plasma Al values were observed only in vegetarians with the CT + TT genotype. The CT + TT genotype was also associated with higher levels of glutamic acid, proline, glycine (only in vegetarians), and aspartic acid.

**Conclusion:**

Although we observed an effect of the *MTHFR* 677C/T polymorphism on folate, certain elements and amino acid levels, further research is required to validate our findings and establish a more comprehensive understanding of the link between elements, amino acids and the *MTHFR* gene polymorphism.

## 1 Introduction

The enzyme methylenetetrahydrofolate reductase (MTHFR) plays a crucial role in the metabolism of folate and one-carbon transfer reactions, as well as in regulating homocysteine levels by reducing 5,10-methylenetetrahydrofolate to 5-methyltetrahydrofolate (5-MTHF), which serves as a donor of the methyl group for homocysteine to methionine conversion. These processes are crucial for DNA methylation. Several polymorphic variants have been identified on *MTHFR* gene, and the most extensively researched variant, located on chromosome 1 (1p36.3), is rs1801133, also known as 677C/T. This mutation leads to a reduction in the MTHFR enzyme activity (1–3), resulting in impaired folate metabolism and an accumulation of homocysteine in the body, causing hyperhomocysteinemia. This condition has been associated with various health issues, including cardiovascular disease, atherosclerosis, and Alzheimer’s disease (2, 4–7).

The influence of the *MTHFR* 677C/T polymorphism on metabolic pathways is complex and can be modulated by a variety of environmental factors, including diet. Adequate consumption of nutrients involved in one-carbon metabolism, particularly folate, vitamin B_12_, and vitamin B_6_, is essential to compensate for reduced enzyme activity in individuals carrying the polymorphism (8–10). The availability of amino acids such as methionine, serine and glycine, which are also part of the one-carbon metabolism, can also be affected by dietary choices (11, 12). Given their different dietary profiles, vegetarianism and non-vegetarianism represent two distinct dietary approaches that require special consideration when analyzing these interactions and their potential consequences on the metabolic implications of the *MTHFR* 677C/T variation. Vegetarian diets are usually rich in plant-based foods, providing abundant amounts of vitamins B_1_, C, E, and folate, along with fibre, antioxidants, polyunsaturated fatty acids and phytochemicals (13–16). However, based on the literature data and our earlier studies, vegetarian diet tends to be lower in proteins, essential amino acids, essential elements such as zinc (Zn), iron (Fe), selenium (Se), iodine (I), and calcium (Ca), as well as vitamins A, D_3_, and particularly B_12_ (17–22). These differences in diet are important, because individuals carrying the *MTHFR* 677C/T mutation may have different metabolic responses depending on their dietary habits, which can have a significant impact on metabolic function and overall metabolic health.

Recent studies, including data from the National Health and Nutrition Examination Survey (NHANES), suggest an association between metals, homocysteine, and folate (23, 24). Toxic elements, particularly lead (Pb), cadmium (Cd) and mercury (Hg), can interfere with homocysteine and folate metabolism by disrupting the normal function of enzymes involved in the one-carbon cycle, a pathway that relies on folate and vitamin B_12_ to regulate homocysteine levels (25). Folate deficiency may heighten the susceptibility of individuals to adverse effects of toxic elements (24, 26–28). In addition, in individuals with reduced folate, Pb was positively associated with homocysteine levels (26, 27), while in boys with lower B_12_ and folate, Hg was negatively correlated with homocysteine levels (29). Other studies have highlighted associations of amino acids with elements and folate, such as their beneficial effects on Zn absorption (30, 31) and the solubility of folic supplements (32). Folate deficiency may lead to higher histidine excretion which can be prevented by folic acid supplementation (33). Conversely, high-dose of folate supplementation can mask anemia, worsen neurological symptoms, and increase cognitive impairment (34). Moreover, vitamin B_12_ deficiency can interfere with methylation-dependent processes, given that methionine synthase uses 5-methyltetrahydrofolate as a one-carbon donor (35).

Prior research examining the influence of polymorphism on health has predominantly concentrated on the specific interaction between polymorphism and individual metabolic components, primarily lipids and folate (36–42), and less frequently amino acids (43–45) or particular essential/toxic substances (46–49). To date, no study has systematically explored the intricate interplay between genetic polymorphisms and the wide range of metabolic markers within a single, integrative framework.

In light of the established relationships between elements, amino acids, and folate, this research aims to investigate the interaction between the *MTHFR* 677C/T polymorphism and dietary habits (vegetarian vs. non-vegetarian), focusing on their combined influence on key biochemical markers. Specifically, it focuses on amino acids that are either essential–must be obtained from diet–or involved in one-carbon metabolism; essential and toxic elements that are influenced by dietary intake and can be reliably measured in blood and/or plasma; and biochemical parameters previously associated with trace elements or *MTHFR* genotype. This integrative approach could provide new insights into the role of personalized nutrition in managing the health risks associated with genetic variations in the *MTHFR* gene.

## 2 Materials and methods

### 2.1 Study population and sample collection

This cross-sectional study included 162 healthy adult participants recruited in Croatia in 2020 and 2021. Each participant was required to sign an informed consent form after they were fully informed about the purpose and methodology of the study. The confidentiality of participants was preserved through the use of unique codes assigned to each sample and study data. The Ethics committee of the Institute for Medical Research and Occupational Health, Zagreb reviewed and approved the study protocols, ensuring compliance with the principles of the Helsinki Declaration. A questionnaire was used to collect general information about age, sex, education, height, weight and socioeconomic status, as well as dietary preferences, smoking status, and other lifestyle habits. The inclusion criteria in this study were as follows: adult vegetarians and non-vegetarians, no clinical signs of acute infection, and for women, an absence of pregnancy or breastfeeding during the study period. The study participants were divided into two groups based on their dietary habits: the vegetarian group, consisting of subjects who were vegan or lacto/ovo-vegetarian (17), and the non-vegetarian group, which included omnivores and pescatarians (those who consumed fish and/or seafood). Pescatarians are classified as non-vegetarians since they consume animal meat, such as fish and seafood, and hence cannot be considered vegetarians (17, 50). Plant-based diets inside the green box should be considered vegetarian, since they avoid meat and seafood.

Blood samples from each participant were collected between 7 and 10 a.m. using special vacutainer tubes (Becton-Dickinson, New Jersey, USA; containing K_2_-EDTA anticoagulant) for trace element analysis. The samples were then aliquoted into clean micro-tubes (Eppendorf, Hamburg, Germany) and stored at +4 °C for elemental analysis, or at −80 °C for DNA isolation. The remaining blood samples were centrifuged for 20 min at 1100 g (Eppendorf, Hamburg, Germany) to separate the plasma, which was then transferred into clean micro-tubes (Eppendorf, Hamburg, Germany), and stored at −80 °C until the element, amino acid, and biochemical parameters analysis. This is especially important in terms of amino acid analysis, because it is well known that pre-analytical factors, such as freeze–thaw cycles, can affect the stability and concentration of amino acids in plasma (51).

### 2.2 Element analysis

The inductively coupled plasma–mass spectrometry (ICP-MS) on an Agilent 8800 (Agilent Technologies, Santa Clara, CA, USA) was used for the analysis of toxic and essential elements in blood/plasma samples. Prior to element analysis, the blood and plasma samples were diluted (1:70 for blood and 1:20 for plasma) using a solution that contained 3 μg L^−1^ of internal standards, 0.01 mM EDTA, 0.7 mM NH_3_, and 0.07% (*v*/*v*) Triton X-100 (52). The working conditions of the ICP-MS, which were optimized with a tune solution of 1 μg L^−1 7^Li, ^59^Co, ^89^Y, ^140^Ce, and ^205^Tl, are detailed in Supplementary Table S1. Sample preparation and analysis were done in the laboratory with a Heating, Ventilating, and Air Conditioning (HVAC) system combined with HEPA filters. To identify potential contamination during the sampling, aliquoting, and dilution of blood and plasma samples, blank samples were also analyzed alongside the real blood and plasma samples. The accuracy of measurement, which ranged from 93 to 113% (Supplementary Tables S2, S3), was verified through the use of commercially available reference materials [Seronorm™ Trace Elements Whole Blood Level I, II and III and Serum Level I and II (Sero AS, Billingstad, Norway), ClinChek® Whole Blood Control Level I, II and III, Serum Level I and II, and Plasma Level I and II (Recipe, Munich, Germany)] and regular participation in the Interlaboratory Comparison Programme UK NEQAS (Birmingham, UK) for Trace Elements in blood and serum.

### 2.3 Biochemical parameters analysis

Biochemical parameters (glucose, triglycerides, total cholesterol, HDL cholesterol, LDL cholesterol, folate, and B_12_ vitamin) in plasma samples were measured by standardized and harmonized methods (Supplementary Table S4) on the Atellica Solution Analyser (Siemens, Erlangen, Germany). The reagents and calibrators were provided by the analyser manufacturer (Siemens, Erlangen, Germany), while quality control samples were obtained from Bio-Rad Laboratories (Hercules, California, USA) for glucose, triglycerides, and total, HDL, and LDL cholesterol, and from Sero (Billingstad, Norway) for B_12_ vitamin and folates. Verification of biochemical parameters analyzed on the Atelica Solution analyzer was performed according to protocols that are compliant with Clinical and Laboratory Standards Institute (CLSI) guidelines and daily imprecision was followed using commercial quality control samples.

### 2.4 Amino acids analysis

Concentrations of 25 amino acids (alanine, sarcosine, glycine, *α*-aminobutyric acid, valine, *β*-aminoisobutyric acid, leucine, allo-*iso*-leucine, *iso*-leucine, threonine, serine, proline, asparagine, aspartic acid, methionine, 4-hydroxyproline, phenylalanine, glutamic acid, *α*-aminoadipic acid, cysteine, ornithine, lysine, histidine, tyrosine, tryptophan) were measured in the plasma samples by Trace 1,300 gas chromatograph (Thermo Scientific, Milan, Italy) coupled to an ITQ 700 ion trap mass spectrometer (Thermo Scientific, Austin, TX, USA). Before GC–MS analysis, samples were processed using the Phenomenex EZ:faast™ amino acid analysis kit as described in Badawy et al. (53). Norvaline was used as an internal standard. Chromatographic separation was performed on a ZB-AAA GC column (10 m × 0.25 mm ID; 0.25 μm film thickness). The injection port was operated in split mode (1:10) at 250 °C. The carrier gas (helium) flow was constant at 1.1 mL/min. The oven temperature program was set at 30 °C/min starting from 100 °C until reaching the final temperature of 320 °C. The operating conditions for the MS system were as follows: electron ionization mode at the energy of 70 eV, transfer line temperature 180 °C, and ion source temperature 240 °C. Full scan mode with a scan range of m/z 45–450 was used for data acquisition.

### 2.5 DNA isolation and genotyping of *MTHFR* 677C/T polymorphisms

DNA was isolated from blood using QIAamp DNA Blood Mini Kit (Qiagen, Hilden, Germany), while BioSpec-nano (Shimadzu, Japan) was used to check the concentration and purity of isolated DNA. The amount of DNA in the eluate, measured by absorbance at 260 and 280 nm, is used to determine the DNA yield. The ratio of absorbance at 260 nm to 280 nm (A260/A280) is used to assess DNA purity. The A260/A280 ratio for pure DNA was in the range from 1.7 to 1.9. After isolation, DNA was stored at −20 °C until polymorphism analysis.

Polymerase chain reaction (PCR) on a T100 thermal cycler (Bio-Rad Laboratories, Hercules, California, USA) was used to amplify the fragment of 198-bp with the following primers: 5′-TGA AGG AGA AGG TGT CTG CGG GA-3′ (forward) and 5′-AGG ACG GTG CGG TGA GAG TG-3′ (reverse). Amplification was conducted under the following conditions: initial denaturation at 95 °C for 5 min, followed by 35 cycles of denaturation at 95 °C for 30 s, annealing at 60 °C for 30 s, and extension at 72 °C for 30 s, with a final extension at 72 °C for 5 min. PCR products were digested with *HinfI* (New England BioLabs, Hitchin, UK) as a restriction enzyme (39, 54) at 37 °C for 4 h. After digestion, the fragments were separated by electrophoresis on 2.5% agarose gel with Lonza GelStar strain (Lonza Group Ltd., Basel, Switzerland) and visualized under an ultraviolet illuminator. The characteristic fragments for the recessive homozygotes genotype (TT) were 175-bp and 23-bp, for the heterozygotes genotype (CT) were 198-bp, 175-bp, and 23-bp, while for the dominant homozygotes genotype (CC) were 198-bp. To confirm the quality of the analysis, 15% of the randomly selected samples were repeated.

### 2.6 Statistical analysis

The concordance between observed allele frequency and Hardy–Weinberg equilibrium was calculated using the *χ*^2^ test, while the differences in genotype frequencies between non-vegetarians and vegetarians were tested by Fisher’s exact test. Study subjects were divided into two main groups based on the *MTHFR* 677C/T polymorphism: the wild-type genotype (CC genotype, *n* = 73) and the CT + TT genotype (*n* = 87). Considering the low frequency of the TT genotype (6.9%), assessing the effects associated with this group would have led to unreliable estimates and greatly reduced statistical power. Therefore, a dominant genetic model (CT + TT *vs*. CC) was used, acknowledging that TT-specific effects might be obscured. Participants were further stratified based on their dietary habits into non-vegetarians (CC genotype *n* = 43; CT + TT genotype *n* = 49) and vegetarians (CC genotype *n* = 30; CT + TT genotype *n* = 38).

Differences between groups based on dietary habits (non-vegetarians *vs.* vegetarians) and genotypes (CT + TT *vs*. CC genotype) were tested by the analysis of variance (ANOVA) and Duncan’s *post-hoc* test. Additionally, the associations between the studied parameters and dietary habit and genotype, adjusted for age, sex, smoking, BMI, education, and intake of dietary supplements, were analysed using multiple regression analyses. Key demographic parameters included age and gender; prospective lifestyle factors involved BMI and smoking status; education level served as a proxy for socioeconomic status; and dietary supplement intake was considered to account for potential side effects. Variables like smoking, sex, education, and supplement intake were considered as categorical. Sex was categorized as male/female, smoking as yes/no, supplement intake was specified by type of supplement (folic acid, vitamin B_12_, minerals, and amino acids), while education was divided into two levels: (1) secondary school *vs*. (2) university degree and post-graduate degree (PhD). Initial models also included the effect of interaction between dietary habit and genotype. In cases when this interaction was not statistically significant, it was omitted from the final model.

For variables with <10% of values below the limit of detection (LOD), values below LOD were set to 0.5 LOD for the subsequent analyses. In cases when more than 10% of values were below LOD (As in blood (63%), Al (41%), *α*-aminoadipic acid (12%), and cysteine (38%) in plasma), differences between groups were tested by ANOVA for censored data, followed by Tukey’s multiple comparison test (55). In the case of cysteine and *α*-aminoadipic acid, differences were tested by the Kruskal–Wallis test (values <LOD are considered as ties) as censored data ANOVA is not recommended in cases when the distribution of residuals deviates from lognormal distribution. For multiple regression analyses, censored likelihood multiple imputation was employed (56). Most of the dependent variables were log-transformed to approach the normal distribution of residuals in ANOVA and regression analysis. The relationships between measured parameters were analysed using Spearman’s correlation coefficients, calculated separately for each combination of genotype (CC and CT + TT genotype) and dietary habit (non-vegetarian and vegetarian). For meaningful interpretation, parameters were grouped based on their nutritional characteristics and biological functions as follows: B vitamins (folates and vitamin B_12_); lipid profile (triglycerides, total cholesterol, HDL, and LDL cholesterol); toxic elements (As, Cd, Hg, Pb, Al, Sn, Tl, Cs); essential elements (Ca, Cu, Fe, I, Mg, Se, Zn, Ba, Sr); trace essential elements (Mo, Mn, Co, Cr); essential amino acids (valine, leucine, isoleucine, threonine, methionine, phenylalanine, lysine, histidine, tryptophan); non-essential amino acids (alanine, glycine, serine, proline, asparagine, aspartic acid, 4-hydroxyproline, glutamic acid, cysteine, ornithine, tyrosine); and non-proteinogenic amino acids (sarcosine, *α*-aminobutyric acid, *β*-aminoisobutyric acid, allo-*iso*-leucine, *α*-aminoadipic acid). Further examining the differences between different types of diets, sparse discriminant analysis (57) was performed. Sparse discriminant analysis (SDA) is a variant of linear discriminant analysis that attempts to find sparse linear combinations of variables that discriminate between different subgroups best. It is especially suitable for limited sample sizes, including situations when the number of variables is larger than the sample size. Furthermore, compared to linear discriminant analysis, the sparseness criterion reduces the number of variables included in each discriminative vector, allowing easier interpretation of results. Differences between three types of dietary habits with respect to measured elements in blood and plasma and amino acids were sought: (1) omnivores (2) lacto-ovo vegetarians, and (3) vegans, while the smallest subgroup (pescatarians; *N* = 10) was excluded from the analysis. The number of variables per each discriminative vector was selected by leave-one-out cross-validation. Before the analysis, all variables were log-transformed and normalized to have equal mean and variance. Variables with more than 10% of values below the LOD (i.e., blood As, plasma Al, *α*-aminoadipic acid, and cysteine) were excluded from the analysis. After selecting the number of variables per discriminative vector by leave-one-out cross-validation, the model was fitted and evaluated on the whole dataset. Thus, the classification accuracy for the whole dataset would correspond to training set accuracy. *Post-hoc* power for detecting gene-diet interactions was calculated based on the F-test for ANOVA model with subgroup sizes of 30 (minimum subgroup size in our sample) and 40 (average subgroup size). Power was calculated for the effect sizes of 0.1, 0.25, and 0.4, corresponding to small, medium, and large effect size, respectively (58). The level of significance was set to 0.05. Power was calculated in G*Power v. 3.1.9.7 (59). Power for a medium effect size of 0.25 was 0.78 for a sample size of 30 per subgroup, and 0.88 for a sample size of 40 per subgroup. For a large effect size of 0.40, the power was larger than 0.99 for both scenarios. On the other hand, the power for detecting interactions with small effect sizes was low (0.19 and 0.24 for the two scenarios).

Statistical analysis was conducted using the software package TIBCO Statistica™, version 14.0.0.15 (TIBCO Software, Inc., USA), and the statistical software R, version 4.3.3 (R Foundation for Statistical Computing, Austria). Multiple regression and ANOVA for censored data were performed in R, as well as sparse discriminant analysis. Package lodi was used for censored likelihood multiple imputation in multiple regression analysis, package NADA2 for censored data ANOVA, while package sparseLDA was used for discriminant analysis. Any results with a *p*-value below 0.05 were considered significant.

## 3 Results

### 3.1 General characteristics of the study group

Table 1 displays the general characteristics [median and interquartile range (IQR)] of the study cohort, as well as the *MTHFR* 677C/T frequency information, while flowchart of participants is presented in Supplementary Figure S1. The median age of the study group was 35 years (range: 18–65 years), and 48.1% of the participants held a university degree, while 32.7% obtained a PhD. Three vegetarian participants did not disclose their educational background. In the study group, 28.4% of subjects were male, while 26.2% smoked cigarettes, with median value of 7 (4–11) cigarettes per day. In terms of dietary habits, 44.3% of vegetarian participants identified as vegans, while 55.7% identified as lacto-ovo vegetarians. They adhered to this diet for 9 years (4–21), with their primary motivations being animal welfare (86% of participants), planet sustainability (74%), health concerns (63%), and to a lesser extent, meat aversion (30%). Out of the participants in the non-vegetarian group, a small percentage (10.9%) followed a pescatarian diet, while the majority (89.1%) were omnivores. Vegetarians had significantly lower (*p* < 0.001) BMI [21.6 (20.2–23.7)] compared to those who consume meat [23.2 (21.7–24.9)]. However, they consumed significantly higher (*p* = 0.011) amounts of supplements (78.6%) in comparison to non-vegetarians (58.7%). Out of these 109 participants taking any type of dietary supplementation, 93 participants were taking vitamin B_12_, minerals, or folic acid, with 13 participants taking all three types of supplements and 27 participants taking two of these three supplements (*N* = 25 for simultaneous intake of minerals and B_12_ and *N* = 2 for minerals and folic acid). No significant differences were found in the allele frequencies between non-vegetarians and vegetarians (*p* = 0.730). Vegetarians exhibited a frequency prevalence of *MTHFR* 677C/T of 44.1% homozygous dominant (CC), 47.1% heterozygous (CT), and 8.8% homozygous recessive (TT), compared to non-vegetarians who had a frequency prevalence of 46.7% CC, 47.8% CT, and 5.5% TT. Allele frequencies were in accordance with the Hardy–Weinberg equilibrium for all participants combined (*χ*^2^ = 2.22, *p* = 0.136), as well as for non-vegetarians (*χ*^2^ = 2.16, *p* = 0.142) and vegetarians (*χ*^2^ = 0.384, *p* = 0.536) considered separately.

**Table 1:** Characteristics of the study participants in relation to dietary habits.

	Non-vegetarians (*n* = 92)	Vegetarians (*n* = 70)	All (*n* = 162)	*p*
General characteristics				
Age (years)	36 (28–41)	34 (27–41)	35 (28–41)	0.308
Education^1^				0.006
Secondary school	14 (15.2)	14 (20.0)	28 (17.3)	
University degree	38 (41.3)	40 (57.1)	78 (48.1)	
Post-graduate degree (PhD)	40 (43.5)	13 (18.6)	53 (32.7)	
Body Mass Index (kg m^−2^)	23.2 (21.7–24.9)	21.6 (20.2–23.7)	22.6 (20.8–24.7)	<0.001
Sex				
Male	26 (28.3)	20 (28.6)	46 (28.4)	1
Female	66 (71.7)	50 (71.4)	116 (71.6)	
Smoking habit				
Smokers	25 (27.2)	18 (25.7)	43 (26.5)	1
Cigarettes per day	9 (5–14)	5 (4–10)	7 (4–11)	
Supplements intake	54 (58.7)	55 (78.6)	109 (67.3)	0.011
Vitamin B_12_	23 (25.0)	45 (64.3)	68 (42.0)	<0.001
Folic acid	6 (6.5)	9 (12.8)	15 (9.2)	0.183
Minerals	37 (40.2)	26 (37.1)	63 (38.9)	0.746
Dietary habit				
Lacto-ovo vegetarianism	/	39 (55.7)		
Veganism	/	31 (44.3)		
Vegetarianism (years)	/	9.0 (4.0–21)		
Frequency of *MTHFR* 677C/T polymorphism (rs1801133)^2^				
Homozygous dominant (CC) genotype	43 (46.7)	30 (44.1)	73 (45.6)	
Heterozygous (CT) genotype	44 (47.8)	32 (47.1)	76 (47.5)	
Homozygous recessive (TT) genotype	5 (5.5)	6 (8.8)	11 (6.9)	
Allele frequency				0.730
C allele	70.7%	67.6%	69.4%	
T allele	29.3%	32.4%	30.6%	

Results are presented as median and IQR (25–75% percentile) or number and percentage (%). ^1^The education level of three vegetarian subjects was not reported. ^2^The *MTHFR* genotype of two vegetarian subjects was not determined. The differences between vegetarians and non-vegetarians were assessed using either the Mann–Whitney’s U-test or the Fisher’s test, with a significance level of *p* < 0.05.

### 3.2 The influence of gene-dietary interaction on the levels of toxic and essential elements

**Figure 1:**
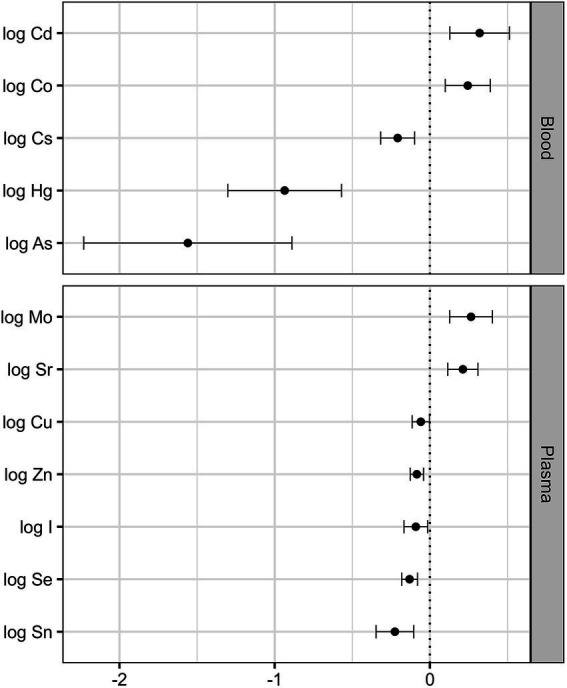
Associations between dietary habits and elements in blood and plasma, represented as regression coefficients and 95% confidence intervals for each element, obtained using multiple linear regression (reference group: non-vegetarians) after adjusting for age, sex, genotype, smoking status, body mass index, level of education, and intake of dietary supplements.

Table 2 shows element concentrations in whole blood and plasma of the non-vegetarians and vegetarians with regard to the *MTHFR* 677C/T polymorphism obtained by ANOVA. Results showed that vegetarians had significantly higher levels of Cd and Co in blood, as well as Sr and Mo in plasma, than non-vegetarians. On the other hand, the levels of several elements in their blood (Cs, Hg, and As) and plasma (Sn, Se, Zn, I, Ca, and Cu) were significantly lower compared to non-vegetarians. Plasma Ca levels were increased in participants with the CT + TT genotype. Additionally, plasma Al levels of vegetarians with the CT + TT genotype were significantly lower than those with the CC genotype (2.00 (1.54–2.96) μg L^−1^ *vs.* 2.68 (1.78–3.91) μg L^−1^, respectively), whereas the plasma Al levels in non-vegetarians with the CT + TT genotype (2.92 (2.32–3.59) μg L^−1^) were comparable to those with the CC genotype (3.08 (2.53–3.43) μg L^−1^). To better understand the associations between the studied parameters and dietary habits and genotype adjusted for potential confounders, we conducted a multiple regression analysis (Figure 1; Supplementary Table S5). While ANOVA analysis indicated a significant interaction between dietary habits and genotype for blood Mn, this effect was not confirmed in multiple regression analysis adjusted for age, sex, smoking, BMI, education, and supplement intake. Interaction between genotype and dietary habits remained statistically significant for Al in plasma (*β* = −0.34 [−0.62, −0.06], *p* = 0.019). Furthermore, multiple regression analysis showed that participants with the CT + TT genotype among both non-vegetarians and vegetarians exhibited a lower blood As (*β* = −0.47 [−0.84, −0.09], *p* = 0.017) and an increased plasma Ca (*β* = 0.01 [0.002, 0.03], *p* = 0.015) compared to those with the CC genotype (Supplementary Table S5). All differences in dietary habits (vegetarians vs. non-vegetarians) observed in the ANOVA analysis, except for plasma Ca, remained significant in the multiple regression analysis, adjusted for age, sex, smoking, BMI, education, and supplement intake, across all measured elements in blood and plasma.

**Table 2:** The concentration of elements in whole blood and plasma of non-vegetarians and vegetarians with regard to the *MTHFR* 677C/T gene polymorphism.

Element	CC genotype		CT+TT genotype	
	Non-vegetarians (*N* = 43)	Vegetarians (*N* = 30)	Non-vegetarians (*N* = 49)	Vegetarians (*N* = 38)
Whole blood				
As (μg L^−1^)^#^	1.54 (0.80–3.92)	0.550 (0.500–0.658)	1.17 (0.663–2.07)	0.578 (0.500–0.669)
Cd (μg L^−1^)^#^	0.521 (0.274–0.845)	0.625 (0.399–0.984)	0.461 (0.353–0.744)	0.658 (0.443–1.21)
Co (μg L^−1^)^#^	0.231 (0.202–0.315)	0.340 (0.270–0.413)	0.274 (0.204–0.399)	0.368 (0.269–0.506)
Cr (μg L^−1^)	0.539 (0.422–0.672)	0.504 (0.456–0.580)	0.517 (0.434–0.674)	0.521 (0.437–0.634)
Cs (μg L^−1^)^#^	3.23 (2.66–4.00)	2.51 (1.82–3.61)	3.59 (2.50–4.31)	2.42 (2.08–2.97)
Hg (μg L^−1^)^#^	1.80 (0.802–4.68)	0.782 (0.288–1.89)	2.32 (0.963–4.24)	1.61 (0.323–2.15)
Mn (μg L^−1^)^*^	8.67 (7.37–10.2)	9.70 (8.11–11.3)	9.38 (7.82–12.1)	9.13 (7.24–10.6)
Pb (μg L^−1^)	9.45 (6.36–18.2)	9.68 (7.61–18.0)	11.8 (9.13–15.0)	11.7 (8.04–19.2)
Tl (μg L^−1^)	0.039 (0.034–0.051)	0.043 (0.034–0.050)	0.039 (0.033–0.045)	0.043 (0.038–0.051)
Plasma				
Al (μg L^−1^)^#,§,*^	3.08 (2.53–3.43)	2.68 (1.78–3.91)	2.92 (2.32–3.59)	2.00 (1.54–2.96)
Ba (μg L^−1^)	0.529 (0.391–0.719)	0.498 (0.384–0.688)	0.455 (0.385–0.551)	0.530 (0.368–0.728)
Ca (mg L^−1^)^#,§^	89.9 (87.9–92.1)	88.9 (86.7–91.9)	91.1 (89.5–94.3)	90.1 (87.2–93.2)
Cu (μg L^−1^)^#^	622 (582–692)	630 (496–689)	659 (575–724)	576 (533–675)
Fe (mg L^−1^)	1.39 (1.09–1.70)	1.34 (0.904–1.71)	1.37 (1.09–1.70)	1.33 (0.875–1.68)
I (μg L^−1^)^#^	39.8 (36.6–48.4)	37.6 (33.4–46.2)	40.4 (34.7–46.1)	35.9 (32.3–43.5)
Li (μg L^−1^)	0.924 (0.601–1.65)	0.765 (0.366–1.49)	0.776 (0.310–1.67)	0.688 (0.329–1.52)
Mg (mg L^−1^)	19.8 (18.9–20.6)	19.3 (18.5–20.2)	20.0 (19.1–20.8)	19.7 (18.7–20.6)
Mo (μg L^−1^)^#^	0.825 (0.665–1.04)	0.912 (0.817–1.37)	0.881 (0.744–1.01)	1.12 (0.858–1.63)
Se (μg L^−1^)^#^	100 (93.6–107)	84.5 (77.8–103)	102 (93.1–111)	88.7 (78.9–97.0)
Sn (μg L^−1^)^#^	0.417 (0.301–0.549)	0.304 (0.227–0.455)	0.366 (0.305–0.493)	0.328 (0.221–0.451)
Sr (μg L^−1^)^#^	20.0 (16.6–21.6)	25.6 (19.2–28.6)	18.9 (15.4–22.5)	23.9 (20.9–29.0)
Zn (μg L^−1^)^#^	776 (696–843)	709 (660–776)	800 (715–842)	733 (689–784)

Results are presented as the median and IQR (25–75% percentile). The differences between vegetarians and non-vegetarians, as well as the CT + TT and CC genotypes, were tested by ANOVA and *post-hoc* Duncan’s test. The exceptions were blood As and plasma Al, which were analysed using ANOVA for censored data and Tukey’s multiple comparison test, as more than 10% of the values were < LOD. The statistical significance of both methods was set at *p* < 0.05. Statistical significance for main effects and interaction are denoted as follows: ^#^Vegetarians vs. non-vegetarians; ^§^CT + TT vs. CC genotype; *Interaction between diet and genotype.

In addition, results of the multiple linear regression analysis (Supplementary Table S5) revealed that the concentrations of Pb, Cs, and As in blood, as well as Cu and Sr in plasma increased with age, with *p*-values ranging from 0.007 to 0.033. The blood Pb (*β* = 0.29 [0.08, 0.51], *p* = 0.007) and plasma Fe (*β* = 0.43 [0.27, 0.58], *p* < 0.001), Mo (*β* = 0.16 [0.008, 0.31], *p* = 0.039), Se (*β* = 0.09 [0.03, 0.14], *p* = 0.003), Ca (*β* = 0.03 [0.01, 0.04], *p* < 0.001), and Sn (*β* = 0.15 [0.02, 0.29], *p* = 0.027) levels in male participants were significantly higher than those in female participants, whereas their blood levels of Mn (*β* = −0.12 [−0.23, −0.02], *p* = 0.023) and Co (*β* = −0.41 [−0.57, −0.24], *p* < 0.001) were significantly lower. Smoking was associated with lower plasma Cu (*β* = −0.07 [−0.13, −0.01], *p* = 0.023) and higher blood Cd (*β* = 0.90 [0.68, 1.10], *p* < 0.001) levels. Participants with increased BMI had higher blood Cs (*β* = 0.02 [0.002, 0.04], *p* = 0.028), while those with higher education levels exhibited lower Cd (*β* = −0.25 [−0.49, −0.005], *p* = 0.045) and higher Cs (*β* = 0.14 [0.004, 0.28], *p* = 0.043) in the blood. The only two elements in plasma that exhibited a negative correlation with supplement intake were Sn (*β* = −0.14 [−0.26, −0.02], *p* = 0.021) and Al (*β* = −0.13 [−0.25, −0.001], *p* = 0.048).

### 3.3 The influence of gene-dietary interaction on biochemical parameters

**Figure 2:**
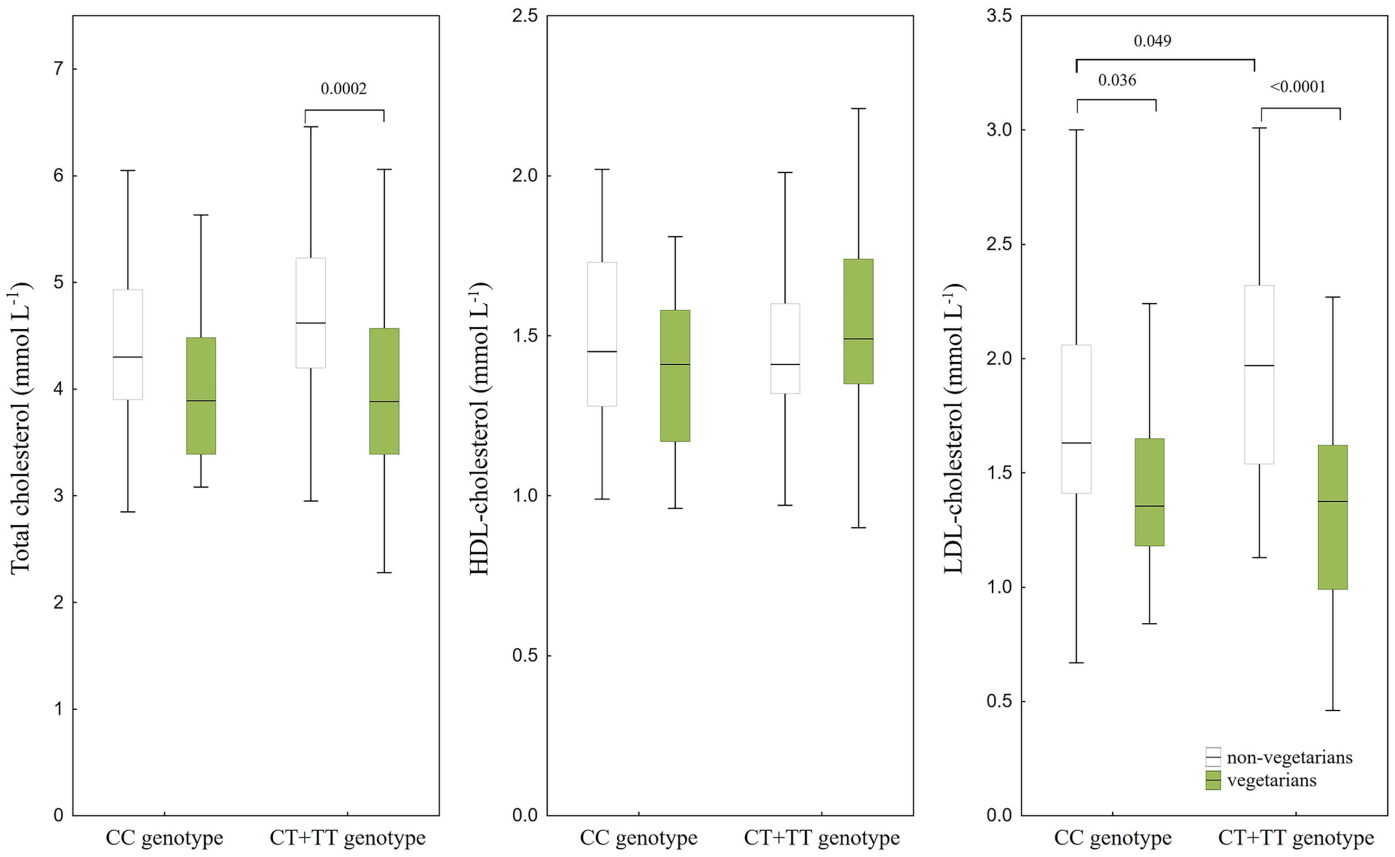
Concentrations of total cholesterol, HDL, and LDL cholesterol in plasma of non-vegetarians (CC genotype *n* = 43; CT + TT genotype *n* = 49) and vegetarians (CC genotype *n* = 30; CT + TT genotype *n* = 38) in relation to *MTHFR* 677C/T polymorphism (CC genotype *n* = 73; CT + TT genotype *n* = 87), presented by box and whisker plots. Only significant differences between genotypes (tested separately within each diet group) and diet (tested separately within each genotype) obtained by ANOVA *post-hoc* comparisons are indicated.

**Figure 3:**
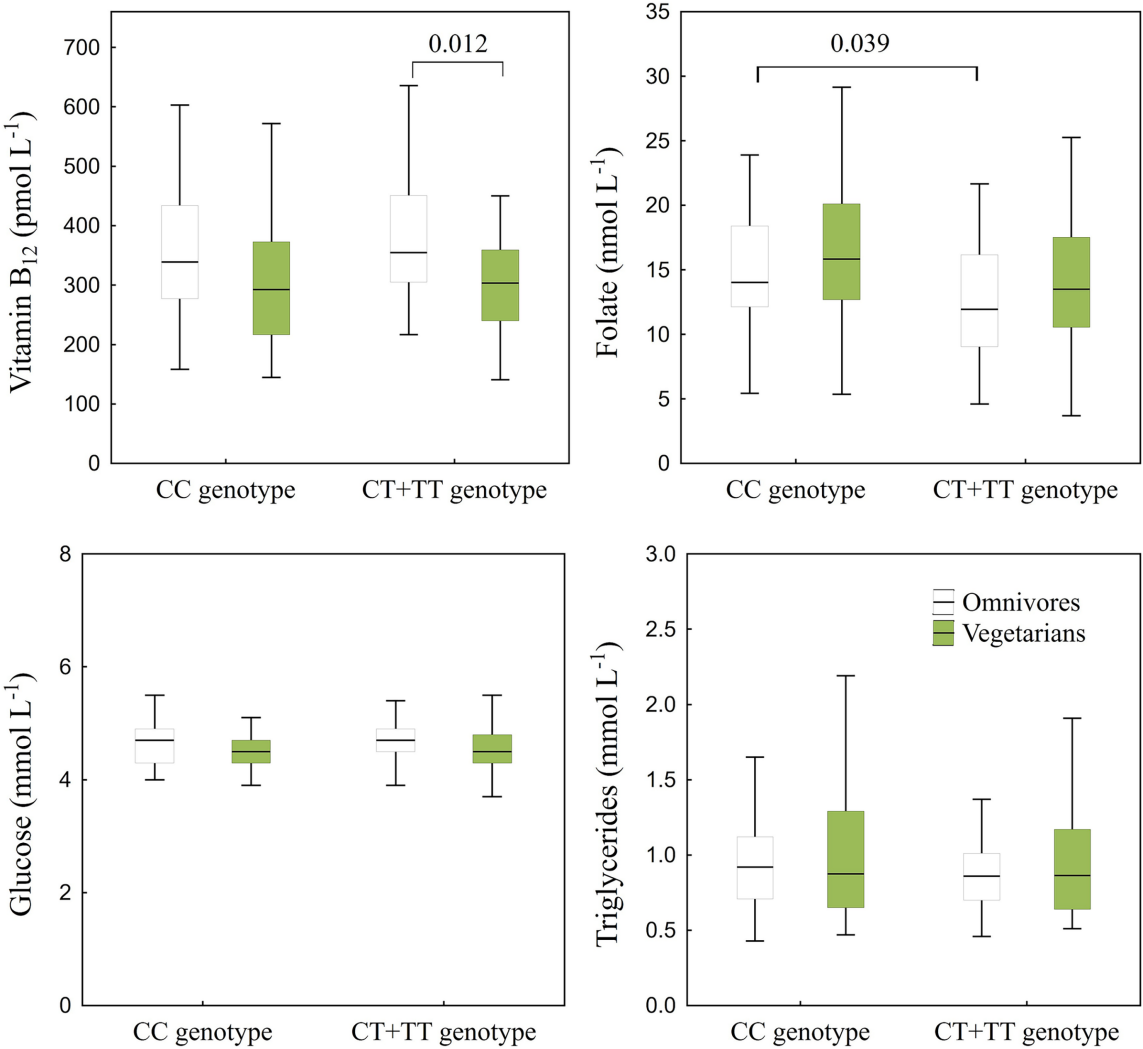
Concentrations of vitamin B_12_, folate, glucose, and triglycerides in plasma of non-vegetarians (CC genotype *n* = 43; CT + TT genotype *n* = 49) and vegetarians (CC genotype *n* = 30; CT + TT genotype *n* = 38) in relation to *MTHFR* 677C/T polymorphism (CC genotype *n* = 73; CT + TT genotype *n* = 87), presented by box and whisker plots. Only significant differences between genotypes (tested separately within each diet group) and diet (tested separately within each genotype) obtained by ANOVA *post-hoc* comparisons are indicated.

The concentrations of biochemical parameters in plasma (total, HDL, and LDL cholesterol, glucose, triglycerides, folate, and vitamin B_12_) in non-vegetarians and vegetarians in relation to the *MTHFR* 677C/T polymorphism are illustrated in Figures 2, 3. The results of ANOVA (Figures 2, 3) showed statistically significant differences in total cholesterol (*p* = 0.0002) and vitamin B_12_ (*p* = 0.012) levels between vegetarians and non-vegetarians in the CT + TT genotype group, with lower levels observed in vegetarians. Reduced levels of LDL cholesterol were found in vegetarians in both genotype groups compared to non-vegetarians (Figure 2). Furthermore, non-vegetarians with the CT + TT genotype had higher levels of LDL cholesterol (*p* = 0.049) and lower levels of folate (*p* = 0.039) than non-vegetarians with the CC genotype. The ANOVA analysis also indicated a statistically significant interaction between diet and genotype only for LDL cholesterol (*F*(1,156) = 5.64, *p* = 0.019), suggesting that non-vegetarian CT + TT genotype had higher LDL cholesterol levels (1.97 (1.54–2.32) mmol L^−1^) than the CC genotype (1.63 (1.4–2.06) mmol L^−1^), whereas LDL cholesterol levels in vegetarians were nearly identical for both genotypes (1.37 (0.99–1.62) mmol L^−1^ for CT + TT genotype and 1.35 (1.18–1.65) mmol L^−1^ for the CC genotype). On the other hand, the results of multiple regression analysis (Supplementary Table S6), adjusted for age, sex, genotype, smoking, BMI, education, and supplement intake, showed a significant genotype-diet interaction only for HDL cholesterol (*β* = 0.16 [0.003, 0.33], *p* = 0.046). Findings from the multiple regression analysis confirm associations between genotype and folate (CT + TT genotype had lower folate levels than CC genotype), although a significant association with LDL cholesterol was not confirmed. Significant associations between diet and total and LDL cholesterol, as well as vitamin B_12_ were confirmed in the multiple regression analysis, while also revealing a negative association with HDL cholesterol (*β* = −0.14 [−0.25, −0.01], *p* = 0.028).

Furthermore, the findings of the multiple regression analysis showed that total and LDL cholesterol increased with age (*β* = 0.01 [0.003, 0.009], *p* = 0.001 for total cholesterol; *β* = 0.01 [0.004, 0.01], *p* = 0.001 for LDL cholesterol), and BMI (*β* = 0.01 [0.004, 0.02], *p* = 0.006 for total cholesterol; *β* = 0.03 [0.01, 0.04], *p* < 0.001 for LDL cholesterol). Increased levels of triglycerides were also associated with higher BMI (*β* = 0.03 [0.009, 0.04], *p* = 0.003), age (*β* = 0.006 [0.0002, 0.01], *p* = 0.043), and smoking (*β* = 0.18 [0.05, 0.31], *p* = 0.007), while glucose was associated only with BMI (*β* = 0.009 [0.005, 0.01], *p* < 0.001). Additionally, vitamin B_12_ was positively associated with supplementary intake (*β* = 0.22 [0.10, 0.34], *p* = 0.001).

### 3.4 The influence of gene-dietary interactions on amino acid levels

**Figure 4:**
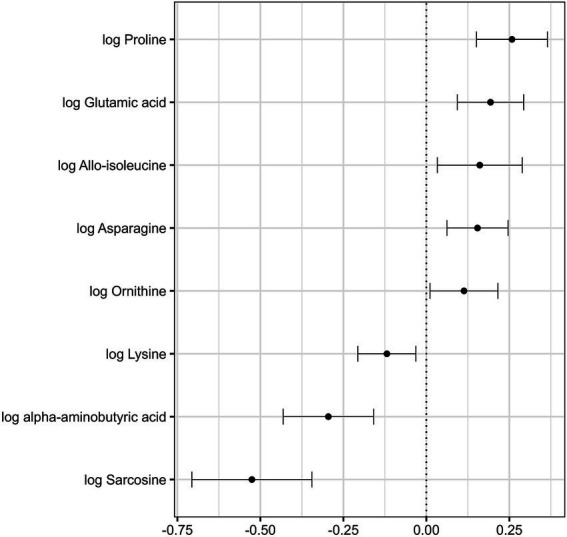
Associations between dietary habits and amino acid concentrations in plasma, represented as regression coefficients and 95% confidence intervals for each amino acid, obtained using multiple linear regression analysis (reference group: non-vegetarians) after adjusting for age, sex, genotype, smoking status, body mass index, level of education, and intake of dietary supplements.

Table 3 shows the concentrations of amino acids in the plasma of vegetarians and non-vegetarians with regard to the *MTHFR* polymorphism. The results of ANOVA (Table 3) showed that vegetarians had reduced levels of sarcosine, *α*-aminobutyric acid, lysine, leucine, 4-hydroxyproline, and higher levels of alanine, proline, glycine, ornithine, allo-*iso*-leucine, asparagine, and glutamic acid in comparison to non-vegetarians. Furthermore, CT + TT genotype had significantly higher levels of proline and glutamic acid than CC genotype. Results of ANOVA (Table 3) also indicated a significant interaction between dietary habits and genotype for asparagine (*F*(1,155) = 4.11, *p* = 0.044), glycine (F(1,155) = 4.59, *p* = 0.034), and alanine (F(1,155) = 4.91, *p* = 0.028), with latter two further confirmed in regression analysis (interaction *β* = 0.16 [0.005, 0.32], *p* = 0.043 and *β* = 0.21 [0.04, 0.37], *p* = 0.013, for glycine and alanine, respectively; Supplementary Table S7). Precisely, plasma levels of alanine, asparagine, and glycine in the CT + TT genotype were higher in vegetarians than in non-vegetarians, while in the CC genotype these differences were not observed. Additionally, multiple regression analysis adjusted for age, sex, smoking, BMI, education, and supplement intake (Figure 4; Supplementary Table S7) confirmed significant effects of dietary habits on sarcosine, *α*-aminobutyric acid, allo-*iso*-leucine, proline, asparagine, 4-hydroxyproline, glutamic acid, ornithine, and lysine, but not on leucine. Although both analyses indicated lower values of 4-hydroxyproline among vegetarians, residuals from the parametric regression model deviated significantly from a normal distribution. Multiple regression analysis confirmed the effects of genotype on glutamic acid and proline, and additionally revealed increased aspartic acid among participants with CT + TT genotype (*β* = 0.20 [0.02, 0.38], *p* = 0.027).

**Table 3:** The concentration of amino acids in plasma of non-vegetarians and vegetarians with regard to the *MTHFR* 677C/T gene polymorphism.

Amino acid (nmol mL^−1^)	CC genotype		CT + TT genotype	
	Non-vegetarians (*N* = 43)	Vegetarians (*N* = 30)	Non-vegetarians (*N* = 48)	Vegetarians (*N* = 38)
Alanine^#,*^	355 (333–425)	396 (315–485)	346 (298–389)	401 (350–492)
Sarcosine^#^	9.69 (7.06–18.5)	6.16 (4.65–7.84)	8.50 (6.12–13.2)	5.70 (4.57–6.54)
Glycine^#,*^	232 (197–274)	252 (210–308)	231 (199–260)	292 (243–341)
*α*-aminobutyric acid^#^	47.1 (35.1–62.4)	33.6 (20.0–47.5)	44.5 (36.2–61.7)	30.3 (25.1–38.8)
Valine	518 (449–602)	501 (433–557)	515 (452–612)	519 (424–591)
*β*-aminoisobutyric acid	4.09 (2.08–5.22)	4.00 (1.96–5.27)	4.33 (1.30–6.0)	3.71 (1.48–4.34)
Leucine^#^	110 (91.4–123)	97.7 (83.0–108)	102 (92.1–121)	103 (83.5–120)
Allo-*iso*-leucine^#^	1.16 (0.865–1.54)	1.38 (1.03–1.60)	1.14 (0.817–1.45)	1.30 (1.01–1.56)
*Iso*-leucine	58.7 (47.4–68.9)	58.1 (48.3–66.9)	54.7 (50.7–72.2)	64.2 (52.7–70.1)
Threonine	104 (69.1–141)	91.2 (64.9–134)	85.3 (63.8–116)	104 (70.0–158)
Serine	100 (73.8–126)	106 (82.8–122)	94.4 (77.7–115)	96.8 (86.01–131)
Proline^#,§^	198 (162–244)	240 (179–272)	200 (164–248)	265 (215–345)
Asparagine^#,*^	45.1 (37.9–57.5)	50.3 (41.6–59.4)	42.1 (36.5–48.5)	56.1 (46.9–68.2)
Aspartic acid	5.26 (3.77–7.78)	6.65 (4.74–9.56)	6.79 (5.05–8.27)	7.15 (4.88–9.52)
Methionine	29.7 (26.5–33.3)	28.4 (25.9–31.3)	28.4 (26.1–31.6)	30.3 (27.0–35.0)
4-hydroxyproline^#^	20.0 (18.5–21.7)	19.6 (18.3–22.0)	20.3 (18.8–22.3)	20.0 (18.4–20.9)
Phenylalanine	60.6 (48.4–68.4)	58.0 (46.9–71.2)	55.7 (43.1–71.3)	65.8 (51.4–83.6)
Glutamic acid^#,§^	45.2 (38.6–54.1)	55.8 (48.0–63.2)	51.5 (42.4–66.5)	55.7 (48.1–69.8)
*α*-aminoadipic acid	3.22 (2.01–4.48)	3.24 (1.79–4.65)	2.89 (1.69–4.96)	2.30 (0.546–3.77)
Cysteine	25.1 (0.293–42.5)	24.0 (0.293–39.4)	26.7 (0.293–43.5)	27.8 (0.293–44.3)
Ornithine^#^	77.2 (68.7–94.7)	93.2 (71.6–115)	79.7 (64.7–91.9)	88.9 (76.0–106)
Lysine^#^	160 (139–209)	144 (122–158)	156 (132–186)	137 (123–163)
Histidine	75.3 (61.5–84.1)	74.5 (57.7–95.7)	63.7 (50.9–77.6)	69.6 (57.9–91.7)
Tyrosine	86.9 (69.1–95.0)	84.1 (63.4–108)	78.5 (57.6–97.8)	84.5 (69.3–102)
Tryptophan	56.9 (47.5–67.3)	55.7 (44.3–66.4)	54.4 (40.6–69.9)	58.0 (42.2–71.5)

Results are presented as median and IQR (25–75%). Differences between vegetarians and non-vegetarians, as well as between the CT + TT and CC genotype, were primarily analysed using ANOVA and *post-hoc* Duncan’s test. As an exception, differences between groups for cysteine and α-aminoadipic acid were tested by Kruskal–Wallis test as over 10% of the values were below the LOD and the distributions did not appear to be lognormal (values < LOD are considered as ties). The statistical significance of both methods was set at *p* < 0.05. Statistical significance for main effects and interaction are denoted as follows: ^#^Vegetarians vs. non-vegetarians; ^§^CT + TT vs. CC genotype; *Interaction between diet and genotype.

Furthermore, regression analysis indicated that the sex of the participants had a notable impact on the levels of measured amino acids. Thus, male participants exhibited higher levels of over half of the measured amino acids (proline, methionine, phenylalanine, glutamic acid, *α*-aminoadipic acid, valine, leucine, allo-*iso*-leucine, *iso*-leucine, *β*-aminoisobutyric acid, ornithine, lysine, tyrosine, and tryptophan) than their female counterparts, with *p*-values ranging from <0.001 to 0.029 (Supplementary Table S7). Increases in serine (*β* = 0.14 [0.01, 0.27], *p* = 0.031), proline (*β* = 0.13 [0.02, 0.25], *p* = 0.020), aspartic acid (*β* = 0.25 [0.04, 0.45], *p* = 0.018), *α*-aminoadipic acid (*β* = 1.10 [0.23, 1.97], *p* = 0.014), glutamic acid (*β* = 0.17 [0.07, 0.28], *p* = 0.002), and ornithine (*β* = 0.11 [0.004, 0.22], *p* = 0.042) were significantly correlated with smoking. The data also revealed a negative association between age and concentrations of leucine (*β* = −0.47 [−0.88, −0.06], *p* = 0.026), while showing positive associations with glutamic acid (*β* = 0.006 [0.0004, 0.01], *p* = 0.036) and ornithine (*β* = 0.005 [0.0001, 0.01], *p* = 0.046). A positive association was observed between BMI and alanine (*β* = 0.02 [0.008, 0.03], *p* = 0.001), *α*-aminobutyric acid (*β* = 0.02 [0.004, 0.04], *p* = 0.019), sarcosine (*β* = 0.03 [0.006, 0.06], *p* = 0.015), and valine (*β* = 0.02 [0.005, 0.03], *p* = 0.003). Additionally, individuals with a high level of education had marginally higher levels of *α*-aminobutyric acid (*β* = 0.17 [0.007, 0.34], *p* = 0.041).

### 3.5 Results of Spearman’s correlation and sparse discriminant analysis

Results of the Spearman’s correlation analysis of all measured parameters are presented as heatmaps of correlation coefficients in Supplementary Figures S2–S5. Overall, positive correlations were more frequent and stronger than negative ones across the entire biomarker panel. The strongest associations were found within biologically related groups, whereas correlations between major biomarker classes (lipids, elements and amino acids) were generally weaker, suggesting largely independent variation across these domains. Within the lipid profile, triglycerides, total cholesterol, LDL and HDL cholesterol showed the expected positive inter-correlations, with the exception of associations of HDL cholesterol with triglycerides and LDL cholesterol. Folate and vitamin B_12_ were weakly related to most other markers, with a positive correlation with each other. Amino acids showed the most pronounced clustering. Essential amino acids, particularly valine, leucine, and *iso*-leucine, were strongly and positively inter-correlated. Non-essential amino acids also displayed positive correlations within their group (e.g., glutamic acid, aspartic acid, serine, and proline) and with essential amino acids. Tyrosine, in particular, displayed high correlations with several essential amino acids (threonine, methionine, phenylalanine, lysine, histidine, and tryptophan). Among elements, several toxic elements were significantly positively correlated (e.g., As with Hg, and Tl with Cd and Pb), whereas negative correlations were observed between selected essential and toxic elements, such as Cd with I, Mg, Se, and Cu.

**Figure 5:**
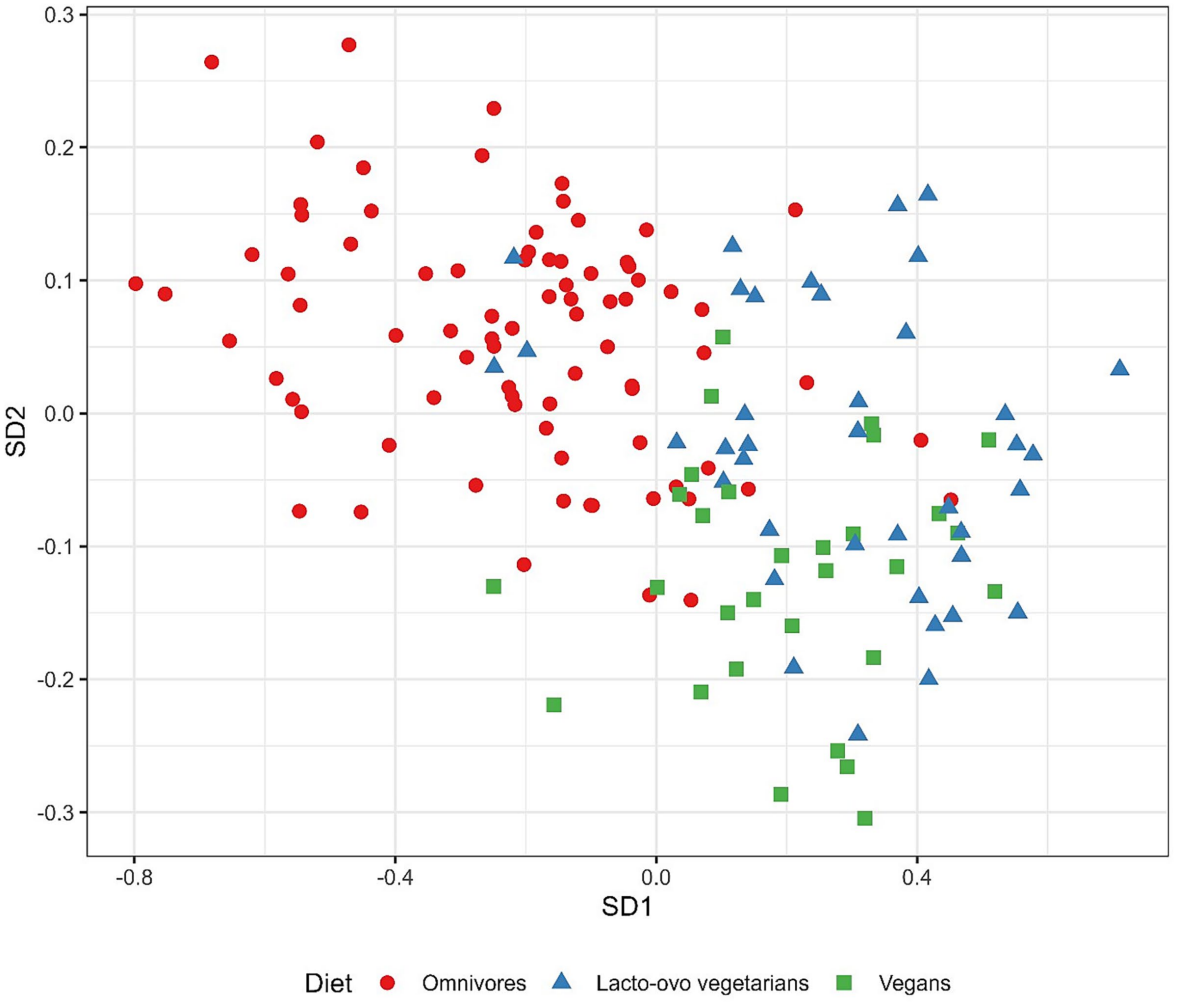
Outcomes of sparse discriminant analysis. The figure shows the projection of data onto the first (SD1) and second (SD2) discriminant vectors. Coefficients for SD1 were as follows: −1.56 (blood Hg), −0.65 (plasma Se), −3.16 (sarcosine), −0.46 (*α*-aminobutyric acid), 0.94 (proline), and 0.45 (asparagine), while coefficients for SD2 were: −0.06 (plasma Mo), 0.84 (plasma Cu), −0.67 (plasma Sr), 0.49 (*α*-aminobutyric acid), −0.04 (*β*-aminoisobutyric acid), and 0.42 (lysine).

Based on the cross-validation procedure, six variables per discriminant vector were selected in order to retain sparseness while keeping the classification accuracy sufficiently high (67%) in comparison to the maximum achieved accuracy (75%), which was achieved when the number of variables per discriminant vector exceeded 38. Figure 5. shows the projection of data on two resulting SDA discriminant vectors, after applying the SDA to the whole dataset. The classification accuracy for the whole dataset was 77%. The first discriminant vector (SD1), effectively discriminates between (1) omnivores and (2) lacto-ovo vegetarians and vegans. Namely, lacto-ovo vegetarians and vegans, predominantly positioned on the right side of the graph, had higher SD1 values (coefficients shown in the figure caption), suggesting that participants with higher levels of proline and asparagine and lower levels of Hg, Se, sarcosine and *α*-aminobutyric acid were more likely to be lacto-ovo vegetarians or vegans. With respect to SD2, the largest difference was noted between omnivores (on average highest values of SD2) and vegans (lowest values of SD2), while lacto-ovo vegetarians were positioned between these two groups (Figure 5). However, the overlap between the groups seemed to be more pronounced compared to SD1. In comparison to an omnivore diet, vegans generally had higher plasma Sr and Mo levels and lower plasma Cu, lysine, and α-aminobutyric acid levels. Regarding *β*-aminoisobutyric acid levels, vegans had, on average, higher values than lacto-ovo vegetarians but lower values than omnivores.

## 4 Discussion

This study investigated the effects of *MTHFR* 677C/T gene polymorphism and dietary habits on the levels of elements and amino acids, and biochemical parameters in a Croatian adult population. According to our findings, vegetarians had better lipid profiles than non-vegetarians, as evidenced by their lower levels of total and LDL-cholesterol (usually referred as “bad cholesterol”). This suggests that vegetarians may be at a lower risk of developing atherosclerotic plaque, which results in the narrowing of blood vessels (60–62). We also found that vegetarians had lower levels of certain trace elements and minerals, such as iodine, selenium, copper, and zinc, than non-vegetarians. This is likely due to the low presence or reduced bioavailability of these nutrients in plant-based diets (63). The patterns observed for lipid profiles and trace elements are consistent with trends observed in our previous study exploring a vegetarian diet (18). Compared to non-vegetarians, vegetarians had higher levels of non-essential amino acids, such as alanine and glycine, while essential amino acid lysine was significantly lower. The variations among the study groups are consistent with the known nutritional makeup of plant-based foods, which typically have lower lysine content but higher glutamate and glycine contents (64). Furthermore, the factors identified by the SDA analysis confirmed that lacto-ovo vegetarians and vegans have higher levels of proline and asparagine, indicating greater intake of plant-based protein, which may be linked to metabolic and oxidative profiles (65, 66). Although there is limited evidence in human populations, reduced levels of sarcosine and *α*-aminobutyric acid may suggest different patterns of amino acid metabolism. Both metabolites have been linked to metabolic dysregulation, gut-brain communication, and oxidative stress (67, 68). At the same time, they have lower levels of blood Hg and plasma Se, indicating that they consume less fish and seafood, which reduces their exposure to Hg but also decreases Se intake, essential for antioxidant enzymes (69, 70). Vegans, in particular, tend to have lower lysine levels than omnivores, a key amino acid linked to impaired protein synthesis (71), and *β*-aminoisobutyric acid, which is important for lipid and glucose regulation, as well as exercise response (72). Their lower levels of plasma Cu indicate reduced bioavailability of Cu from plant-based foods (73), and elevated levels of plasma Sr and Mo likely reflect higher intake of plant-based foods and water (74, 75). In light of the observed differences in the biochemical data, element, and amino acid profiles between vegetarians and non-vegetarians, the study participants had to be categorized according to their eating preferences. This approach reduced the possibility of bias from dietary differences while enabling us to more thoroughly investigate the effects of genetic polymorphism.

### 4.1 Genotypic distribution and allelic frequencies

It is well established that both geographical location and ethnicity have a significant impact on the frequency of the homozygous recessive (TT) genotype of *MTHFR* 677C/T gene polymorphism. The prevalence of this genotype worldwide is the highest among Europeans (34.1%), followed by populations in Australia (20.5%), Asia (19.7%), South America (27.8%), North America (31.2%), Africa (10.3%), and Middle East (7.5%). On the other hand, the lowest prevalence of TT genotype was reported for people of African descent (0–1.2%) (2, 10, 76). A study conducted on Mexican Amerindians reported that geographical and ethnic factors influence the prevalence of the *MTHFR* 677C/T polymorphism. The prevalence in the Southeastern region ranged from 23 to 71%, followed by the Southern region (0.08 to 68%) and the Central Eastern region (0 to 57%), while the lowest frequencies were observed in Mexican Amerindians from the Northern region (0 to 19%) (77). In Europe, the lowest frequency of TT genotype was recorded in Germany (7.8%), followed by the Netherlands (8.9%), Norway (9.4%), France (9.8%), Sweden (10.3%), and Ireland (10.8%), while the highest prevalence was observed in Britain (13.2%) and Italy (18%) (76). The genotype frequencies obtained in this study (45.6% of CC, 47.5% of CT, and 6.9% of TT) are in agreement with the global occurrence of the TT genotype (10), and are comparable to those found in earlier studies conducted in the Croatian population (78–81).

### 4.2 The effects of the *MTHFR* gene polymorphism on folate and B_12_ levels

*MTHFR* 677C/T polymorphism is responsible for a 50–60% decrease of MTHFR activity in individuals with the homozygous recessive (TT) genotype compared to the wild-type (CC) genotype, leading to a decreased production of 5-MTHF from folate. This, in turn, impairs the conversion of homocysteine to methionine, resulting in elevated homocysteine levels and hyperhomocysteinemia (2). In the absence of sufficient levels of vitamin B_12_ and folate, which are crucial cofactors in this reaction, the body will accumulate homocysteine. A study of the German population with varying dietary habits found that vegetarians with homozygous recessive genotype (TT) had slightly lower (*p* = 0.06) serum folate levels than vegetarians with wild-type genotype (CC) (82). The association between the TT genotype and lower folate levels in comparison to those with the CC genotype has also been observed in study of the Chinese hypertensive population (83). In our study, CT + TT genotype exhibited significantly lower (ANOVA *F*(1,156) = 7.8, *p* = 0.006) plasma folate levels than those with the CC genotype. These results were also supported by the results of multiple regression analysis (*β* for genotype: −0.19 [−0.32, −0.06], *p* = 0.005; Supplementary Table S6). Furthermore, with a deeper *post-hoc* analysis we observed that non-vegetarians with the CT + TT genotype exhibited significantly lower (*p* = 0.039) plasma folate levels (11.9 (9.06–16.2) nmol L^−1^) than those with the CC genotype (14.0 (12.1–18.4) nmol L^−1^). Similarly, vegetarians with CT + TT genotype had lower folate levels (13.5 (10.6–17.5) nmol L^−1^) in comparison to those with the CC genotype (15.8 (12.7–20.1) nmol L^−1^), although the difference was not statistically significant (*p* = 0.094), which might be explained by lower statistical power in *post-hoc* tests. Interestingly, when obtained values were compared to the cut-off value for folate deficiency defined by the manufacturer [<7.63 nmol L^−1^ (84)], we discovered that a lower percentage of individuals with the CC genotype were folate deficient (6.9% of non-vegetarians and 3.3% of vegetarians) compared to those with CT and TT genotypes (14.3% of non-vegetarians and 7.9% of vegetarians, respectively). These results suggest that individuals with the CT + TT genotype may be more susceptible to hyperhomocysteinemia caused by folate deficiency than those with the wild-type (CC) genotype. However, the design of our study does not allow us to draw firm conclusions regarding the impact of the *MTHFR* 677C/T polymorphism on folate metabolism, as homocysteine levels, a key downstream biomarker in the 5-MTHF metabolic pathway, were not measured. Therefore, we cannot determine whether the observed differences are caused directly by disruptions in folate and homocysteine metabolism or result from other mechanisms linked to the *MTHFR* variant, such as epigenetic, neurochemical, oxidative, vascular, or gene–nutrient pathways. According to the literature, the only known mechanism responsible for intracellular activation of all forms of folate to 5-MTHF is MTHFR (2, 85). 5-MTHF serves as a methyl donor for the conversion of homocysteine to methionine and as a precursor to S-adenosylmethionine (SAM), which is further a cofactor or donor of methyl groups for DNA methyltransferases (86). A polymorphism in the *MTHFR* gene causes instability and reduces enzymatic activity, which leads to decreased methyl folate synthesis. This, in turn, diminishes one-carbon metabolism required for DNA synthesis and methylation (85, 87). Additionally, a meta-analysis of folic acid intervention studies revealed that individuals with the TT genotype had significantly lower folate status than CC/CT and CC genotypes (41). Surprisingly, these differences in folate status between genotypes persisted even after a minimum of 8 weeks of folate supplementation, despite an increase in mean serum folate levels, suggesting that individuals with TT genotype did not respond as expected to folic acid supplementation (41). According to a German population study, TT individuals with low folate or vitamin B_12_ status had a higher risk of hyperhomocysteinemia than those with the CC genotype, who have high levels of both folate and B_12_ (82). Lower plasma folate and vitamin B_12_ levels in TT genotype with the mild folate-deficiency was reported by Pereira et al. (88). Opposite to this finding, we did not detect any differences between the genotypes for vitamin B_12_ and this disparity between our and Pereira’ study can likely be attributed to variations in individuals’ supplementation to prevent B_12_ deficiency. For instance, 42% of our participants took vitamin B_12_ supplements. However, we are unable to make this assertion with certainty, since Pereira et al. (88) did not provide any information regarding vitamin B_12_ supplementation. Vitamin B_12_ deficiency results in lower remethylation of homocysteine and production of *S*-adenosylmethionine, which in turn affects the methyltransferase activity. Additionally, 5-MTHF becomes trapped between MTHFR and inhibited methionine synthase, rendering it incapable of participating in the remethylation pathway (89, 90).

### 4.3 The effects of the *MTHFR* gene polymorphism on lipid status

Numerous studies have explored the association between *MTHFR* 677C/T polymorphism and lipid levels, but the findings have been inconsistent (36, 38, 39, 42). Some authors (91, 92) suggested that these inconsistencies could be an indirect consequence of homocysteine mediation or modifications in methylation status, given that MTHFR is an important enzyme in the one-carbon metabolism, wherein 5-MTHF serves as an important methyl donor for various molecules, including homocysteine, DNA, and proteins (93). However, other factors like ethnicity, age, sex, and health status, may also modulate the relationship between lipids and polymorphism, contributing to the variability in findings (39, 42, 94, 95). For instance, while some studies have been unable to establish a correlation between the *MTHFR* 677C/T polymorphism and lipid status in the overall study population, differences became apparent when data were stratified by sex. In several studies, only female with the CT + TT genotype had higher levels of triglycerides and LDL cholesterol in comparison to those with the CC genotype (39, 94). In contrast, Zhang et al. (95) found that, in their study of two distinct Chinese ethnic groups, only males with the CT + TT genotype had higher levels of both LDL cholesterol and triglycerides when data were stratified by sex, although HDL cholesterol levels did not differ by genotypes. In our study, which focused on population stratified by diet, we found a significantly higher (*p* = 0.049) LDL cholesterol level of non-vegetarians with CT + TT genotype (1.97 (1.54–2.32) nmol L^−1^) in comparison to those with the CC genotype (1.63 (1.41–2.06) nmol L^−1^). These ANOVA results indicated higher LDL cholesterol in non-vegetarians with the *MTHFR* 677C/T polymorphism, suggesting that *MTHFR* 677C/T could be a risk factor for increasing LDL cholesterol in plasma. However, these results did not remain significant in the regression analysis after adjusting for potential confounders, implying that the apparent effect is context-dependent and likely influenced by confounding. Nevertheless, several biologically plausible pathways link dyslipidemia to *MTHFR*-related hyperhomocysteinemia. Elevated homocysteine can activate transcription factors [nuclear factor Y (NF-Y), cyclic adenosine monophosphate response element-binding protein (CREB), sterol regulatory element-binding protein 2 (SREBP-2)], increasing mRNA expression and activity of HMG-CoA reductase, promoting hepatic lipid accumulation, and ultimately contributing to hypercholesterolemia (96). In another pathway, elevated homocysteine can induce endoplasmic reticulum stress in hepatocytes, endothelial cells, and arterial smooth muscle cells, triggering the unfolded protein response and SREBP pathways and up-regulating genes involved in cholesterol synthesis and uptake, as well as the intracellular cholesterol acumulation (97). The loss of statistical significance after adjustment emphasizes the complexity of the relationships between *MTHFR* 677C/T and lipids, which are probably influenced by other metabolic modifiers and nutritional status. However, given the mechanisms described above, our initial ANOVA findings have biological plausibility. A limitation of the current study is that plasma homocysteine levels were not measured, which prevents a proper and logical explanation of the relationship between the examined polymorphism and serum lipid levels. Additionally, significant interaction between diet and genotype was observed for HDL cholesterol after adjustment for age, sex, smoking, BMI and education, indicating opposing trends among vegetarians (lower HDL values for CC genotype) and non-vegetarians (higher HDL for CC genotype). Diet and eating habits are well-recognized important factors that influence gene-lipid interactions. While our study design does not allow us to draw conclusions about mechanisms or pathways, the presence of this interaction suggests that the *MTHFR* 677C/T polymorphism can influence HDL cholesterol levels depending on dietary habits, especially among vegetarians and non-vegetarians. A plausible explanation is that MTHFR activity influences homocysteine metabolism and methylation processes, which in turn regulates lipid metabolism (98, 99). Carriers of the *MTHFR* 677C/T polymorphism exhibit significantly reduced MTHFR enzyme activity and elevated homocysteine levels (10, 100). Associated increased oxidative stress has been linked to elevated homocysteine and decreased expression of transport proteins like ATP-binding cassette transporter A1 (ABCA1), impairing HDL biogenesis (101, 102). Reduced levels of SAM in individuals with the *MTHFR* 677C/T polymorphism may also reduce methylation of genes involved in lipoprotein metabolism, such as apolipoprotein A-1 (APOA1) and lecithin-cholesterol acyltransferase (LCAT) (10, 103, 104). Moreover, it has been reported that many nutraceuticals, such as dietary supplements, can influence lipid profiles and dyslipidemia, potentially exerting a greater effect than individual genetic differences (105, 106).

Although we did not perform analyses stratified by sex, our regression models were adjusted for this variable and showed that male sex was not a significant predictor of plasma lipid status. A recent systematic review and meta-analysis by Luo et al. (42) reported that individuals with the CT and TT genotypes had higher levels of total and LDL cholesterol compared to those with the CC genotype. However, no association was found with triglycerides and HDL cholesterol. Interestingly, when data were stratified by sex and ethnicity, it became evident that the obtained associations were specific to female individuals and were predominantly observed in the Asian population. Gender differences may partly result from women consuming more folic acid than men. This, in turn, affects the increase in homocysteine levels and the differences among genotypes, leading to higher homocysteine levels and increased lipid production in those with the *MTHFR* 677C/T polymorphism (107). Sex-specific associations between lipid plasma levels and *MTHFR* 677C/T polymorphism have also been observed in studies of a long-lived Chinese cohort (39) and an overweight Chinese Han population (38). However, caution is needed when interpreting results of latter study, as Zhi et al. (38) found no significant correlation between the *MTHFR* 677C/T polymorphism and lipid levels in healthy Han individuals with normal BMI. Conversely, obesity and dyslipidaemia are known to be associated with elevated levels of triglycerides, total cholesterol, and LDL cholesterol, as well as a reduction in HDL cholesterol (108). This suggests that the effect attributed to the polymorphism on lipid levels may have been caused by obesity or dyslipidaemia, rather than the polymorphism itself, as these effects were not present in the healthy control group, indicating that the impact of *MTHFR* polymorphisms on lipid levels also depends on the health status of the population studied. In another study conducted by Yin et al. (109), both males and females with the CT + TT genotype and normal weight had higher levels of total, HDL, and LDL cholesterol in comparison to those with the CC genotype. On the other hand, overweight individuals with the CT + TT genotype had higher HDL cholesterol and triglyceride levels, but lower total and LDL cholesterol levels, than the CC genotype. Interestingly, a study of hypertensive individuals (110) found no significant association between the *MTHFR* 677C/T polymorphism and any blood lipid levels. The interaction between the health status of the population and lipid levels was also confirmed in studies that exclusively examined a single sex with specific health conditions, leading to variable results. Therefore, in a study of obese female adolescents, no significant differences in lipid parameters were observed between genotypes (111), whereas women with polycystic ovarian syndrome (PCOS) carrying the CT genotype exhibited higher triglyceride and cholesterol levels compared to those with the CC genotype (112).

### 4.4 The effects of the *MTHFR* gene polymorphism on element levels

Unlike biochemical markers, there is a scarcity of research that investigates the relationship between the *MTHFR* 677C/T polymorphism and element levels in the body. To the best of our knowledge, this study is among the few to examine the effects of this polymorphism on both toxic and essential elements (see Joneidi et al. (25) for a review).

The only literature currently available that investigates the link between the *MTHFR* 677C/T polymorphism and essential elements is limited to Fe, as no evidence was found linking other essential elements to this gene polymorphism. Bortolus et al. (46) investigated the effects of four one-carbon metabolism-related polymorphisms, including *MTHFR* 677C/T, on the Fe and ferritin status of healthy Italian men and women. They found no correlation between the investigated parameters, which is in accordance with our findings regarding the total Fe content in plasma. In addition, we observed significantly higher plasma Ca levels in individuals with the CT + TT genotype (*β* = 0.01 [0.003, 0.03], *p* = 0.015; Supplementary Table S5). This association raises an interesting question about the possible link between this genetic variant and the regulation of Ca homeostasis, although direct evidence connecting *MTHFR 677C/T* polymorphism to circulating Ca is, to our knowledge, lacking. Prior meta-analysis has reported associations between the *MTHFR* 677C/T and bone mineral density (113) as well as fracture risk (114). Because abnormalities in Ca metabolism are associated with the risk of osteopenia and osteoporosis (115) and excessive vascular Ca accumulation is associated with elevated cardiovascular risk (116), the observed genotype-related difference in plasma Ca may have clinical relevance. As mentioned above, *MTHFR* 677C/T polymorphism affects folate and homocysteine metabolism. Mendelian-randomization analyses by Casas et al. (117), who observed that the *MTHFR* T allele carriers have an increased risk of stroke, consistent with allele-related elevations in homocysteine concentration, supports a causal link between elevated homocysteine and stroke risk. It is therefore possible that the T allele, through mechanisms that still need to be clarified, influences various aspects of Ca metabolism with potential impacts on bone and vascular health. Given these considerations, our finding of higher plasma Ca in CT + TT genotype emphasize the need for long-term studies that can evaluate the clinical effects of these changes on the risk of osteoporosis and cardiovascular disease, as well as for functional studies examining the expression of Ca transport proteins and ion channels in relation to the *MTHFR* genotype.

In the present study, we observed that participants with the CT + TT genotype had lower levels of As in their blood than individuals with the CC genotype (*β* = −0.47 [−0.84, −0.09], *p* = 0.017; Supplementary Table S5), suggesting reduced susceptibility to As exposure through food. Nevertheless, we were unable to conclusively confirm this finding, as we employed blood as a biological indicator of exposure to arsenic from food, primarily from fish and seafood, which predominantly contain organic arsenic compounds that are less toxic than inorganic As (iAs) (118, 119). In contrast, most previous studies examining As metabolism in relation to *MTHFR* 677C/T polymorphism focused on exposure to iAs by analyzing its metabolites in urine samples. It is widely recognized that the *MTHFR* 677C/T polymorphism may lead to a decrease in methylation capacity. In the event of human exposure to inorganic As, this may lead to the incomplete methylation of monomethylarsonic acid (MMA) to dimethylarsinic acid (DMA), thereby increasing the risk of As-induced cancer (120, 121). According to a study conducted on populations from Hungary, Romania, and Slovakia (122), which investigated polymorphism and other factors influencing the metabolism of iAs at low environmental levels, males with the CT + TT genotype had a lower proportion of dimethylarsinic acid (%DMA) and a higher percentage of monomethylarsonic acid (%MMA) in their urine than those who were homozygous for the wild-type allele (CC). This suggests that the CT + TT genotype is more susceptible to the accumulation of iAs in the body and an increased occurrence of toxic effects caused by iAs in comparison to the CC genotype. Similar results were observed in Indigenous women from Argentina who were also exposed to iAs through water (123).

The *MTHFR* 677C/T polymorphism has been associated with several other potentially toxic elements. Saad-Hussein et al. (49) found that occupationally exposed workers with the TT genotype had higher urinary Pb concentrations than those with other genotypes. They hypothesized that this might be due to decreased bone mineral density, a condition that has been linked to *MTHFR* gene polymorphisms (124). However, Golbahar et al. (125) failed to establish a correlation between the *MTHFR* 677C/T polymorphism and bone mineral density in postmenopausal women, despite directly measuring bone density. In this study, both vegetarians and non-vegetarians with CT + TT genotype had slightly higher blood Pb levels in comparison to those with the CC genotype, although the difference was not statistically significant. Liu et al. (47) reported that CT and TT with higher Cd levels had an increased risk of neural tube defects. Although the authors did not discuss the relationship between Cd levels and *MTHFR* polymorphism, the presented proportions of new-borns with high Cd levels did not show a statistically significant difference between the CC and CT/TT genotypes. In a study of Inuit population, Parajuli et al. (48) reported that individuals with the CT + TT genotype had lower Hg levels compared to those with the CC genotype. By contrast, our findings indicated slightly higher Hg levels in participants with the CT + TT genotype compared to the CC genotype, although this difference was not statistically significant. The observed discrepancy between studies could stem from the traditionally higher exposure of the Inuit population to Hg through fish and seafood consumption, leading to 2–5 times higher blood Hg levels than those recorded in the Croatian population [this study; (52, 126, 127)] and 16 times higher than in the average Canadian adult (128). However, these conclusions are limited by the lack of supplementary information from Parajuli et al. (48) regarding potential exposure sources, including fish and seafood intake, which may influence Hg levels (129–131). Nevertheless, it is important to recognize that, in addition to the potential harmful impact of Hg from seafood on health, a moderate fish consumption may also have a beneficial role in maintaining DNA integrity (132).

Our results of analysis of variance revealed that plasma Al levels in vegetarians with CT + TT genotype were significantly lower in comparison to the CC genotype. This suggests that vegetarians with the CT + TT genotype might accumulate lower Al levels in the body in comparison to the homozygous CC genotype. This is a novel observation not previously documented in the literature, but it should be interpreted with caution since it was not observed in non-vegetarians. This finding may be of interest in light of recent reports linking the presence of Al in the body (urine and/or hair) with symptoms of autism spectrum disorder (ASD) in children (133–135). In parallel, a recent systematic review and meta-analysis (136, 137) indicated a significant association between the *MTHFR* 677C/T polymorphism and elevated ASD risk; nevertheless, these results should be interpreted cautiously due to small sample sizes and heterogeneity in study characteristics (e.g., ethnicity, genotyping methods, etc.). Furthermore, our study did not assess ASD outcomes and we cannot infer any relationship between plasma Al levels, dietary habits, *MTHFR* 677C/T polymorphism, and ASD. We also did not examine the expression of metallothioneins or other metal-binding proteins that could influence Al bioavailability. Although our results suggest that a vegetarian diet potentially reduces the risk of neurotoxic effects of Al through higher intake of antioxidants that reduce oxidative stress and bind metals, inadequate intake of vitamin B_12_ (crucial for methylation and neutralization of homocysteine) as well as suboptimal intake of other key nutrients, such as Zn, Fe, and omega-3 fatty acids, could also increase the risk in individuals with the *MTHFR* polymorphism. Further studies are needed to elucidate the molecular mechanisms behind this association. It is plausible that different mechanisms are involved – for example, *MTHFR* 677C/T polymorphism may increase the risk for autism through methylation disorders and folate metabolism, independent of Al, while Al act as an additional or separate risk factor that accumulates differently across different genotypic backgrounds. To better understand these relationships, further research should focus on the relevant molecular pathways. Since there is growing evidence that both genetic and environmental factors contribute to ASD, these fascinating findings encourage combined research in this area, which could improve our understanding of the mechanisms that may link exposure to Al and the *MTHFR* 677C/T polymorphism to ASD (138). In this regard, our findings could be helpful in the development of epidemiologic studies that examine the interaction between *MTHFR* polymorphism and the level of Al in the body and their influence on human health. More broadly, our findings raise the question of potential interactions between the *MTHFR* 677C/T polymorphism and exposure to environmental toxins, such as toxic metals that cause oxidative stress and interfere with enzyme activity. In T allele carriers, exposure to heavy metals may worsen metabolic imbalances, as the *MTHFR* 677C/T polymorphism already reduces the availability of 5-methyltetrahydrofolate and elevates levels of homocysteine. Individual differences in sensitivity to metal poisoning may partly reflect gene–environment interactions. To clarify mechanism and clinical relevance, future studies should integrate genetic predisposition with detailed heavy metal exposure assessment. If carriers of specific genetic variants such as *MTHFR* 677C/T are confirmed to have increased vulnerability to environmental toxins, including metals, this could guide exposure-prevention strategies and support the development of personalized public health approaches.

### 4.5 The effects of *MTHFR* gene polymorphism on amino acids

For many years, it has been demonstrated that small variations in the activity of certain enzymes can lead to minor modifications in metabolic fluxes, but can have substantial effects on metabolite concentrations (139). Merging metabolomics and genetic data is necessary to understand the interplay of genes, environment, and diet (140). This investigation involved a metabolomic analysis of plasma samples to identify the amino acids that could distinguish between individuals with and without the *MTHFR* 677C/T polymorphisms. We observed that participants with CT + TT genotype had elevated levels of proline, glutamic acid, and aspartic acid in comparison to the CC genotype, while glycine was elevated only in vegetarians with the CT + TT genotype. The literature on metabolomic changes associated with the *MTHFR* polymorphism is scarce, particularly with respect to variations in amino acids. In the Swedish National Study on Aging and Care, the *MTHFR* 677C/T polymorphism was associated with the homocysteine-to-methionine ratio, with lower levels in the TT genotype compared to the CC genotype. However, no differences were observed in the levels of methionine. Additionally, the research showed that the CT and TT genotypes with low methionine levels were more susceptible to developing cardiovascular multimorbidity than individuals with the CC genotype (45). In this study, methionine levels did not differ across the genotypes. However, the levels of glycine in vegetarians with the CT + TT genotype were significantly higher than those in the CC genotype, suggesting that vegetarians with CT + TT genotype may be more prone to mild hyperhomocysteinemia than those with the CC genotype. The results of our research on the adult population can be partially compared to those of a Spanish study conducted on cerebral creatine transporter deficiency patients, who underwent a regimen of oral glycine and L-arginine therapy administered twice daily over a period of 9 months. The authors observed that patients with the CT + TT genotype exhibited varying degrees of hyperhomocysteinemia, with the severity of the condition increasing with increasing dosages of glycine and L-arginine (43). It is well-established that the protein glycine *N*-methyltransferase (GNMT) is linked with MTHFR by *S*-adenosylmethionine (SAM). When SAM levels are elevated, MTHFR is inhibited, which in turn reduces 5-MTHF, thereby enabling GNMT to reduce SAM levels. Conversely, reduced SAM levels lead to higher production of 5-MTHF, which inhibits GNMT and spares methyl groups for DNA methylation (141–145). A recent study conducted on pregnant women reported that women with *MTHFR* polymorphism had lower levels of 1-monohexadecanoylglycerol, pyrophosphate, benzoin, and linoleic acid, and higher levels of glyceric acid, L-tryptophan, L-alanine, L-proline, norvaline, L-threonine, and myo-inositol than women without polymorphism (44). Our finding of higher proline levels in the CT + TT genotype agrees with their findings. Nevertheless, it is important to take caution when interpreting these data, as the authors did not specify whether they investigated *MTHFR* 677C/T or 1298A/C polymorphism, and their data was based on a small sample size of only 18 women (10 without and 8 with *MTHFR* polymorphism). Proline is essential for the tendons and joints, but it can also act as a neurotoxin and metabotoxin in certain circumstances (65).

As previously mentioned, the literature contains a limited number of studies that examine the relationship between human amino acid profiles and *MTHFR* polymorphisms. To the best of our knowledge, this study is the first to report the significant relationship between this polymorphism and glutamic and aspartic acid in humans. The sole study in the literature that addresses a similar subject is a pilot study conducted on 67 individuals diagnosed with schizophrenia (146), which utilized the homeostatic model assessment of insulin resistance (HOMA-IR) as a marker of cardiovascular disease. Unfortunately, while the authors identified the link between HOMA-IR and two combined *MTHFR* polymorphisms (677C/T and 1298A/C), as well as the associations between HOMA-IR and specific amino acids (a negative correlation with glycine, serine, and betaine, and a positive correlation with glutamate and threonine), they did not explore the relationship between amino acid profiles and *MTHFR* polymorphisms. We also observed significant gene-diet interactions for asparagine, alanine, and glycine, with the latter two remaining significant after multivariate regression analysis adjustment. Although the observational design of our study does not permit causal inference about possible biological mechanisms underlying this interaction, these patterns may be partly explained by their involvement in one-carbon metabolism and amino acid interconversion pathways. For instance, serine hydroxymethyltransferase (SHMT) uses 5,10-methylenetetrahydrofolate for the conversion of glycine into serine, which is generated by the cytosolic nicotinamide adenine dinucleotide phosphate (NADPH)-dependent activity of MTHFR (11, 147). These pathways may be significantly impacted by dietary habits (vegetarian vs. non-vegetarian), which differ in the quantity and quality of micronutrients and protein. These considerations provide testable hypotheses for the observed interactions; their confirmation will require genotype-stratified studies integrating amino acid intakes and one-carbon metabolism biomarkers to clarify the link between *MTHFR* polymorphism and amino-acid metabolism.

### 4.6 Study strengths and limitations

This study is one of the few that offers new insights into the impact of *MTHFR* genetic variants on an individual’s sensitivity to metals and the amino acid profile in human plasma. This is of relevance when assessing how one-carbon-related genetic variations might affect both element and amino acid status. An important strength of this study is the similarity in lifestyle and anthropometric traits between the two dietary groups, which helps to minimize any bias caused by differences in population characteristics. Additionally, the data linking dietary habits with element and amino acid levels, alongside biochemical parameters, may be valuable for designing future studies targeting specific populations, particularly those with individual sensitivities.

However, several limitations should be addressed when interpreting these findings. First, due to the low prevalence rate of the TT genotype (~7%) in our study group (*n* = 162), we were unable to assess its independent effects on the measured variables reliably. Analyzing the TT genotype separately would have substantially reduced the power of statistical tests and increased the risk of generating potentially misleading results. While adequate statistical power for detecting interactions of medium to large effect sizes was maintained in the dominant-model approach, grouping TT and CT genotypes might have obscured the TT-specific effects and introduced bias in interpreting the role of the TT variant. Furthermore, the study was underpowered for detecting interactions of small effect sizes. Second, although the study aimed to include a sample broadly representative of Croatian adults (vegetarians and non-vegetarians), full representativeness across all sociodemographic strata could not be ensured. The use of convenience sampling approach introduces the risk of selection bias. Furthermore, the high percentage of participants with doctoral degrees (particularly in the non-vegetarian group) may indicate a higher level of health awareness and health-oriented behavior compared to the general population, which could have affected their dietary preferences, supplement use and metabolic profiles. Third, our study group was unbalanced in terms of sex (72% females *vs*. 28% males), which may limit the generalizability of the results. However, this distribution aligns with global trends showing higher prevalence of vegetarian dietary patterns among women (148), and a greater willingness of women to participate in research (149, 150). Although regression models were adjusted for sex, the smaller sample size for male participants reduced power to detect sex-specific effects. Furthermore, the interaction effects for sex were not assessed. Future studies should include larger, more diverse, and sex-balanced populations to improve the external validity of the findings. Additionally, since the study was conducted within a single cohort, the results might not be generalizable to other populations with different dietary habits, lifestyle factors/behaviors. Despite these limitations, our research offers new integrative data linking dietary practices, the *MTHFR* gene, and several biochemical markers within a real-life cohort. Fourth, no urine samples were obtained, which prevented us to quantify the levels of iAs metabolites (monomethylarsonic acid, MMA, and dimethylarsinic acid, DMA). Consequently, we could not assess methylation capacity of the CT + TT genotype in comparison to those with the CC genotype. Fifth, we were unable to determine plasma homocysteine levels due to limitations in the study’s design and inadequate sample preparation for homocysteine analysis, thereby preventing us to assess well established relationship between homocysteine and the *MTHFR 677C/T* polymorphism (41, 151). Although we assessed homocysteine levels in a previous study, in which we found that vegetarians had higher levels than non-vegetarian subjects (12.30 ± 3.80 μmol L^−1^ vs. 10.70 ± 2.60 μmol L^−1^) (18), these data could not be integrated with the current dataset or directly compared due to differences in study design and study population. Consequently, we cannot conclusively determine whether the observed differences between the analyzed indicators are mediated by homocysteine or by mechanisms independent of homocysteine. Thus, our understanding of the mechanistic connection between the *MTHFR* genotype and observed effects remains limited. To examine the direct and indirect effects of *MTHFR* on biochemical parameters, elements, and amino acid profiles, future studies should include homocysteine in the analytical panel.

Sixth, aside from the use of dietary supplements, complete information on dietary habits, such as total calorie intake, distribution of macronutrients, and specific nutrient intake, was not included in the analysis. Instead, a broad classification (vegetarian *vs*. non-vegetarian) was used to reflect dietary habits. This limited our ability to capture the full range of individual dietary patterns and nutrient intake and may have constrained interpretation of interactions between *MTHFR* polymorphisms, diet, and biochemical outcomes. A detailed dietary assessment within this cohort is ongoing and will be addressed in future analysis. In addition to diet, various external factors, including water quality, environmental contamination, fish and seafood intake, and occupational exposure, can also influence the levels of both toxic and essential elements in blood and/or plasma. However, as the current study did not analyse these data on these non-dietary exposures, our ability to fully distinguish non-dietary environmental influences from nutritional determinants of elemental status is limited. Seventh, the classification accuracy for sparse discriminant analysis was evaluated on the same dataset that was used for fitting the model. Thus, the classification accuracy on an independent dataset is expected to be lower. Independent evaluation on a new sample is recommended in future studies. Lastly, potential sources of bias and confounding factors must be considered. The high proportion of individuals with PhD, the sex imbalance, and the convenience sampling could have influenced the results. Unmeasured factors such as physical activity, environmental exposures, and micronutrient intake may also distort the observed associations. Additionally, since the results are based on a Croatian population, the findings might not be widely applicable to other groups due to differences in dietary habits, lifestyle choices, and environmental factors.

## 5 Conclusion

In conclusion, this study demonstrates the significant impact of dietary habits on most of the analysed parameters and offers new insights into the relationship between the *MTHFR* 677C/T polymorphism and folate levels, showing that individuals with this polymorphism had lower folate levels. The study also found associations between the *MTHFR* 677C/T polymorphism and plasma Al and Ca levels, and identified novel relationships with the amino acids proline, glycine, aspartic acid, and glutamic acid. Overall, our results primarily highlight the significance of gene–diet interactions associated with trace elements, biochemical parameters, and amino acid profiles, underscoring the necessity for additional study. Although these findings may contribute to developing more personalized dietary plans over time, replication in larger, genotype-stratified cohorts and clinical validation will be required before translation into practice. To better distinguish between the respective contributions of gene-diet and gene–environment interactions, future studies should include more detailed dietary intake data and relevant environmental exposure data.

## Data Availability

The data that support the findings of this study are available from the corresponding authors, upon reasonable request.
