# Supplementary material for: MTHFR 677C/T gene polymorphism and dietary habits: effects on trace element levels, amino acids, and biochemical parameters
Source: Front Nutr. 2026 Jan 12;12:1710613. doi: 10.3389/fnut.2025.1710613 (PMC12832494; doi:10.3389/fnut.2025.1710613)
Supplement: Supplementary file 1 [file Data_Sheet_1.PDF]

Supplementary materials

Frontiers in Nutrition

Tatjana Orct[1,†], Jelena Kovačić[1,†], Ines Peremin[2], Zorana Kljaković-Gašpić[1], Daria Pašalić[3], Ankica Sekovanić[1,*], Adrijana Dorotić[4], Blanka Tariba Lovaković[1], Andreja Jurič[1], Alica Pizent[1], Fran Crnjac[3], Lora Dukić[4,5], Marko Gerić[1], Ivone Jakaša[2], Goran Gajski[1,*]

[1]Institute for Medical Research and Occupational Health, Zagreb, Croatia
[2]University of Zagreb, Faculty of Food Technology and Biotechnology, Zagreb, Croatia
[3]University of Zagreb, School of Medicine, Zagreb, Croatia
[4]University Hospital Sveti Duh, Zagreb, Croatia
[5]Clinical Hospital Center Rijeka, Rijeka, Croatia

[†]These authors contributed equally to this work and share first authorship

* Corresponding authors:

Ankica Sekovanić
Institute for Medical Research and Occupational Health, Zagreb, Croatia
Email: asekovanic@imi.hr

Goran Gajski
Institute for Medical Research and Occupational Health, Zagreb, Croatia
Email: ggajski@imi.hr

Supplementary material contains: 5 Figure and 7 Tables

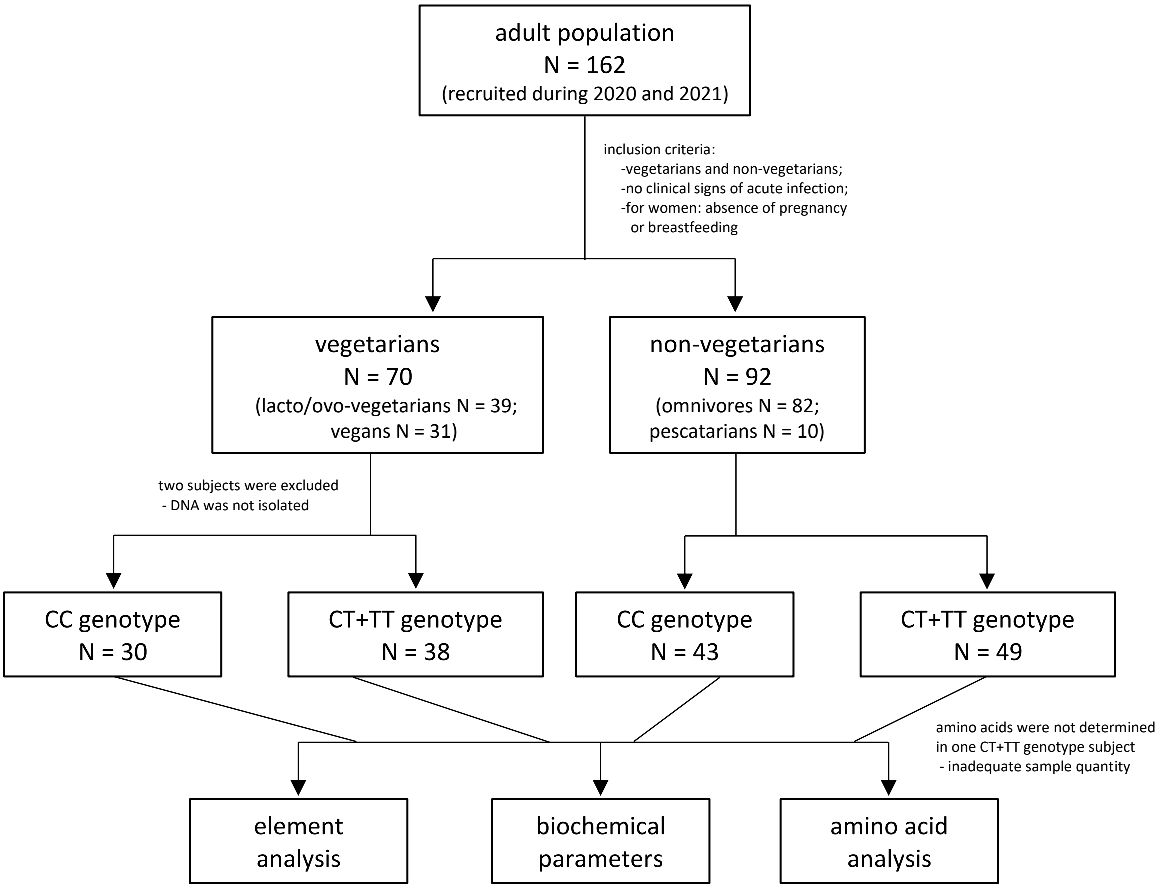

Fig. S1. Flowchart of participants for the current study

Table S1. ICP-MS Agilent 8800 (Agilent Technologies, Santa Clara, CA, USA) working conditions

**Parameter**				
RF Power	1550 W			
RF matching	1.8 V			
Sampling depth	8.0 mm			
Torch-H	-0.4 mm			
Torch-V	0.2 mm			
Nebulizer pump	0.10 rps			
Plasma gas flow rate	15 L/min			
Makeup gas flow rate	0.1 L/min			
Nebulizer gas flow rate	1.05 L/min			
Nebulizer	MicroMist (quartz)			
Spray chamber	Scott type (quartz), cooled at 2°C			
Sample cone	Nickel, 1 mm orifice diameter			
Skimmer cone	Nickel, 0.4 mm orifice diameter			
Doubly-charged ions and oxides limits	$^{140}Ce^{2+}/^{140}Ce^{+}$<0.90 %; $^{140}Ce^{16}O^{+}/^{140}Ce^{+}$<1.23 %			
	**no gas**	**H_2**	**He**	**O_2**
Scan Type	MS/MS	MS/MS	MS/MS	MS/MS
Collision/reaction gas flow rate	/	6.0 mL/min	8 mL/min	30%
Extract lens 1 voltage	0 V	0 V	0 V	0 V
Extract lens 2 voltage	-200 V	-180 V	-160 V	-180 V
Isotopes measured	^{27}Al, ^{202}Hg, ^{205}Tl,	^{54}Fe, ^{78}Se	^{24}Mg, ^{43}Ca, ^{55}Mn, ^{59}Co, ^{63}Cu, ^{68}Zn, ^{88}Sr, ^{95}Mo, ^{111}Cd, ^{118}Sn, ^{127}I, ^{133}Cs, ^{138}Ba, ^{208}Pb,	^{68}Cr, ^{91}As

MS/MS – tandem mass spectrometry

Table S2. Certificated and obtained values of analyzed elements in whole blood

Element	Seronorm™ Trace Elements Whole Blood L I		Seronorm™ Trace Elements Whole Blood L II		Seronorm™ Trace Elements Whole Blood L III	
	Certified (mean (95% CI))	Measured (mean±SD)	Certified (mean (95% CI))	Measured (mean±SD)	Certified (mean (accept. range))	Measured (mean±SD)
As (µg/L)	4.6 (3.7-5.5)	4.8±0.20	14.1 (11.3-17.0)	13.7±0.92	30.4 (23.1-37.7)	30.5±2.68
Ba (µg/L)	457*	464±19.4	477*	469±16.6	700*	697±4.7
Cd (µg/L)	0.28 (0.17-0.40)	0.27±0.020	5.01 (4.00-6.02)	5.37±0.365	12.1 (10.8-13.4)	12.2±0.23
Co (µg/L)	0.20 (0.12-0.28)	0.21±0.042	5.18 (4.13-6.22)	5.39±0.303	11.4 (10.2-12.6)	11.8±0.40
Cr (µg/L)	0.45 (0.27-0.63)	0.48±0.164	10.7 (8.5-12.8)	11.2±1.34	23.2 (18.5-27.9)	26.6±2.95
Cs (µg/L)	2.4*	2.5±0.05	2.3*	2.4±0.07	2.8*	2.7±0.12
Hg (µg/L)	1.48 (1.18-1.77)	1.43	17.0 (13.6-20.4)	18.3±0.64	37.1 (29.6-44.6)	36.2±1.00
Mn (µg/L)	18.4 (14.7-22.1)	18.4±0,17	31.4 (25.1-37.7)	32.3±0.83	47.3 (37.8-56.8)	47.8±0.41
Mo (µg/L)	0.53 (0.41-0.61)	0.51±0.113	5.31 (4.24-6.37)	4.97±0.525	7.5 (6.0-9.0)	7.3±0.16
Pb (µg/L)	9.9 (7.9-11.9)	10.8±0.12	337 (269-405)	333±0.6	447 (401-493)	449±14.7
Tl (µg/L)	0.007 (0.003-0.011)	0.007±0.0027	10.2 (8.1-12.2)	10.7±0.58	34.1 (27.2-41.0)	34.0±2.73
**Element**	**ClinChek® Whole Blood Control L I**		**ClinChek® Whole Blood Control L II**		**ClinChek® Whole Blood Control L III**	
	Certified (mean (Control range))	Measured (mean±SD)	Certified (mean (Control range))	Measured (mean±SD)	Certified (mean (Control range))	Measured (mean±SD)
As (µg/L)	5.42 (4.34-6.51)	5.60±0.136	9.97 (7.97-12.0)	10.29±0.498	19.4 (915.5-23.2)	20.1±0.72
Ba (µg/L)	/	1.02±0.082	/	1.00±0.078	/	0.83±0.089
Cd (µg/L)	1.23 (0987-1.48)	1.32±0.018	2.88 (2.30-3.45)	3.24±0.135	6.32 (5.06-7.59)	6.53±0.236
Co (µg/L)	1.56 (1.24-1.87)	1.57±0.142	7.13 (5.70-8.55)	6.97±0.519	13.1 (10.4-15.7)	13.0±0.94
Cr (µg/L)	1.38 (1.03-7.72)	1.39±0.253	5.61 (4.20-7.01)	5.55±0.304	10.6 (8.44-12.7)	11.1±1.12
Cs (µg/L)	/	2.9±0.27	/	2.8±0.22	/	2.9±0.12
Hg (µg/L)	1.26 (0.885-1.64)	1.36±0.061	6.87 (5.15-8.59)	7.39±0.304	10.7 (8.59-12.9)	10.6±0.41
Mn (µg/L)	8.87 (7.09-10.6)	8.26±0.366	15.4 (12.3-18.5)	14.4±0.14	22.1 (17.7-26.5)	21.2±1.10
Mo (µg/L)	2.07 (1.65-2.48)	2.28±0.167	4.48 (3.58-5.37)	4.76±0.141	8.71 (6.97-10.5)	9.05±0.284
Pb (µg/L)	54.5 (43.6-65.3)	55.6±3.34	219 (176-263)	212±9.7	425 (340-510)	419±5.6
Tl (µg/L)	0.820 (0.656-0.984)	0.832±0.0576	4.19 (3.35-5.03)	4.01±0.423	8.38 (6.70-10.1)	8.51±0.421

*approximate value

Table S3. Certificated and obtained values of analyzed elements in plasma/serum

Element	Seronorm™ Trace Elements Serum L I		Seronorm™ Trace Elements Serum L II	
	Certified (mean (95% CI))	Measured (mean±SD)	Certified (mean (95% CI))	Measured (mean±SD)
Al (µg/L)	33.6 (31.7-35.5)	34.0±1.34	104 (98-110)	104±2.1
Ba (µg/L)	126*	126±6.0	140*	140±22.6
Ca (mg/L)	94.2 (89.8-98.6)	94.6±0.50	145 (137-153)	140±5.9
Cs (ng/L)	18.6*	18.4±0.61	24*	25±6.0
Cu (µg/L)	1691 (1607-1775)	1661±70.5	2887 (2788-2986)	2861±95.3
Fe (mg/L)	1.39 (1.31-1.47)	1.45±0.045	2.03 (1.90-2.16)	2.05±0.047
I (µg/L)	84*	84.4±4.30	89*	83.1±4.96
Li (µg/L)	5741 (5420-6062)	5523±20.1	10950 (9981-11919)	10819±244.0
Mg (mg/L)	20.1 (18.8-21.4)	20.4±0.57	40.8 (36.1-45.5)	40.3±0.20
Mn (µg/L)	15.0 (14.1-15.9)	14.7±0.16	19.9 (18.8-21.0)	19.4±0.28
Mo (µg/L)	0.7*	0.8±0.03	0.9*	0.9±0.07
Se (µg/L)	107 (100-114)	110±2.1	163 (153-173)	158±7.1
Sn (µg/L)	0.52*	0.49±0.016	0.51*	0.49±0.019
Sr (µg/L)	26.3*	26.1±0.38	36.3*	35.1±1.18
Zn (µg/L)	1738 (1667-1809)	1663±91.2	2520 (2314-2726)	2404±86.2
**Element**	**ClinChek® Serum Control L I**		**ClinChek® Serum Control L II**	
	Certified (mean (Control range))	Measured (mean±SD)	Certified (mean (Control range))	Measured (mean±SD)
Al (µg/L)	16.5 (11.5-24.4)	16.1±0.97	60.1 (48.1-72.1)	60.0±1.02
Ba (µg/L)	428 (364-492)	422±14.9	556 (473-640)	557±19.7
Ca (mg/L)	/	56.6±5.65	/	54.7±4.43
Cs (µg/L)	/	0.45±0.024	/	0.46±0.013
Cu (mg/L)	1.06 (0.902-1.22)	1.03±0.018	1.39 (1.18-1.60)	1.34±0.010
Fe (mg/L)	1.09 (0.874-1.31)	1.17±0.040	1.73 (1.38-2.07)	1.84±0.021
I (µg/L)	105 (83.9-126)	103±2.7	149 (119-179)	147±6.2
Li (mg/L)	3.64 (3.27-4.00)	3.77±0.228	7.18 (6.46-7.89)	7.59±0.225
Mg (mg/L)	15.6 (14.0-17.1)	15.7±0.81	20.4 (18.3-22.4)	20.9±1.61
Mn (µg/L)	2.26 (1.81-2.72)	2.21±0.18	6.65 (5.32-7.98)	6.50±0.12
Mo (µg/L)	2.16 (1.51-2.81)	2.11±0.057	4.18 (2.93-5.44)	4.06±0.301
Se (µg/L)	123 (98.1-147)	125±2.5	158 (126-189)	166±5.0
Sn (µg/L)	2.47 (1.97-2.96)	2.54±0.19	18.8 (15.0-22.5)	18.4±0.76
Sr (µg/L)	/	25.4±0.85	/	24.5±0.86
Zn (mg/L)	0.737 (0.626-0.848)	0.799±0.0826	1.09 (0.926-1.25)	1.16±0.109
**Element**	**ClinChek® Plasma Control L I**		**ClinChek® Plasma Control L II**	
	Certified (mean (Control range))	Measured (mean±SD)	Certified (mean (Control range))	Measured (mean±SD)
Al (µg/L)	57.9 (46.3-69.5)	59.9±2.33	92.2 (73.8-111)	93.5±3.31
Ba (µg/L)	727 (618-836)	751±26.7	794 (675-913)	788±32.1
Ca (mg/L)	/	73.7±5.42	/	69.7±4.14
Cs (µg/L)	/	0.59±0.022	/	0.55±0.061
Cu (mg/L)	0.733 (0.623-0.843)	0.698±0.0310	1.26 (1.08-1.45)	1.19±0.037
Fe (mg/L)	0.885 (0.752-1.02)	0.879±0.0363	1.24 (1.05-1.42)	1.27±0.048
I (µg/L)	47.0 (37.6-56.4)	46.6±1.65	99.9 (80.0-120)	98.3±6.32
Li (mg/L)	3.14 (2.83-3.46)	3.27±0.101	8.34 (7.51-9.18)	8.18±0.872
Mg (mg/L)	15.8 (14.2-17.4)	15.7±0.41	29.1 (26.2-32.0)	29.4±0.56
Mn (µg/L)	4.58 (3.67-5.50)	4.42±0.021	15.3 (12.2-18.3)	15.5±0.58
Mo (µg/L)	1.91 (1.34-2.48)	1.84±0.094	6.58 (5.27-7.90)	6.27±0.683
Se (µg/L)	73.8 (59.0-88.5)	75.9±2.99	120 (96.1-144)	124±5.2
Sn (µg/L)	1.15 (0.804-1.49)	1.18±0.089	7.59 (6.07-9.10)	7.49±0.240
Sr (µg/L)	/	41.7±0.45	/	41.5±0.81
Zn (mg/L)	1.76 (1.50-2.02)	1.81±0.127	2.13 (1.81-2.45)	2.12±0.085

*approximate value

Table S4 List of methods used in biochemical analysis

**Analyte**	**Method**
Glucose	Enzymatic method with hexokinase
Triglyceride	Enzymatic method with glycerol-phosphate oxidase and peroxidase
Total cholesterol	Enzymatic method with esterase, cholesterol oxidase and peroxidase
HDL cholesterol	Enzymatic method with elimination chylomicrons, VLDL and LDL lipoproteins
LDL cholesterol	Enzymatic method with elimination all non-LDL lipoproteins
Folate	Competitive direct chemiluminescent (CLIA) immunoassay
Vitamin B_{12}	Competitive direct chemiluminescent (CLIA) immunoassay

Table S5 Results of multiple regression analysis for element levels in blood and plasma

	Intercept	Vegetarian diet	Genotype*	Age	Male sex	Smoking	Body mass index	Education level*	Supplements intake	Adjusted R^2; model p-value§
**Blood:**										
log Hg	0.84	**-0.94<br>[-1.30, -0.57]<br>p < 0.001**	0.06<br>[-0.30, 0.41]<br>p = 0.754	0.01<br>[-0.01, 0.03]<br>p = 0.374	0.25<br>[-0.15, 0.66]<br>p = 0.223	0.09<br>[-0.32, 0.49]<br>p = 0.677	-0.04<br>[-0.09, 0.02]<br>p = 0.175	0.26<br>[-0.20, 0.72]<br>p = 0.271	0.30<br>[-0.07, 0.66]<br>p = 0.109	0.14<br>p < 0.001
log Pb	1.31	0.11<br>[-0.09, 0.30]<br>p = 0.288	0.11<br>[-0.08, 0.292]<br>p = 0.270	**0.01<br>[0.004, 0.02]<br>p = 0.007**	**0.29<br>[0.08, 0.51]<br>p = 0.007**	0.10<br>[-0.12, 0.31]<br>p = 0.370	0.02<br>[-0.01, 0.05]<br>p = 0.193	-0.06<br>[-0.30, 0.19]<br>p = 0.655	-0.02<br>[-0.21, 0.17]<br>p = 0.835	0.09<br>p = 0.003
log Cd	-0.83	**0.32<br>[0.13, 0.51]<br>p = 0.001**	0.05<br>[-0.14, 0.23]<br>p = 0.626	0.01<br>[0.00, 0.02]<br>p = 0.050	-0.19<br>[-0.41, 0.02]<br>p = 0.073	**0.90<br>[0.68, 1.10]<br>p < 0.001**	-0.008<br>[-0.04, 0.02]<br>p = 0.608	**-0.25<br>[-0.49, -0.005]<br>p = 0.045**	-0.10<br>[-0.28, 0.09]<br>p = 0.325	0.35<br>p < 0.001
log As	-0.96	**-1.56<br>[-2.23, -0.89]<br>p = 0.007**	**-0.47<br>[-0.84, -0.09]<br>p = 0.017**	**0.03<br>[0.002, 0.05]<br>p = 0.033**	0.51<br>[-0.13, 1.14]<br>p = 0.107	0.21<br>[-0.20, 0.62]<br>p = 0.301	-0.02<br>[-0.08, 0.04]<br>p = 0.5260	0.84<br>[-0.10, 1.78]<br>p = 0.073	-0.02<br>[-0.48, 0.44]<br>p = 0.9244	
log Tl	-3.65	0.08<br>[-0.03, 0.20]<br>p = 0.158	-0.03<br>[-0.14, 0.08]<br>p = 0.604	-0.001<br>[-0.007, 0.005]<br>p = 0.782	-0.02<br>[-0.14, 0.11]<br>p = 0.797	0.07<br>[-0.06, 0.20]<br>p = 0.290	0.02<br>[0.0004, 0.04]<br>p = 0.056	0.08<br>[-0.06, 0.23]<br>p = 0.261	-0.03<br>[-0.14, 0.08]<br>p = 0.609	0<br>p = 0.511
log Mn	2.56	-0.04<br>[-0.14, 0.05]<br>p = 0.388	0.04<br>[-0.05, 0.13]<br>p = 0.379	0.0002<br>[-0.005, 0.005]<br>p = 0.932	**-0.12<br>[-0.23, - 0.02]<br>p = 0.023**	-0.10<br>[-0.21, 0.001]<br>p = 0.051	-0.01<br>[-0.03, 0.003]<br>p = 0.123	-0.04<br>[-0.16, 0.09]<br>p = 0.574	0.02<br>[-0.07, 0.12]<br>p = 0.644	0.05<br>p = 0.039
log Co	-1.01	**0.24<br>[0.10, 0.39]<br>p = 0.001**	0.10<br>[-0.04, 0.24]<br>p = 0.149	-0.0002<br>[-0.008, 0.008]<br>p = 0.964	**-0.41<br>[-0.57, -0.24]<br>p < 0.001**	-0.11<br>[-0.27, 0.04]<br>p = 0.163	-0.006<br>[-0.03, 0.02]<br>p = 0.582	-0.004<br>[-0.19, 0.18]<br>p = 0.961	0.03<br>[-0.11, 0.17]<br>p = 0.675	0.21<br>p < 0.001
log Cr	0.64	-0.02<br>[-0.07, 0.04]<br>p =0.615	0.005<br>[-0.05, 0.06]<br>p = 0.854	-0.002<br>[-0.005, 0.001]<br>p = 0.180	-0.04<br>[-0.10, 0.03]<br>p = 0.249	-0.006<br>[-0.07, 0.06]<br>p = 0.858	-0.002<br>[-0.01, 0.007]<br>p = 0.728	0.03<br>[-0.04, 0.10]<br>p = 0.466	0.04<br>[-0.02, 0.10]<br>p = 0.189	0<br>p=0.691
log Cs	0.38	**-0.21<br>[-0.32, -0.10]<br>p < 0.001**	-0.007<br>[-0.11, 0.10]<br>p = 0.892	**0.007<br>[0.0008, 0.01]<br>p = 0.027**	0.05<br>[-0.07, 0.18]<br>p = 0.382	0.03<br>[-0.09, 0.15]<br>p = 0.5859	**0.02<br>[0.002, 0.04]<br>p = 0.028**	**0.14<br>[0.004, 0.28]<br>p = 0.043**	0.02<br>[-0.08, 0.13]<br>p = 0.660	0.20<br>p < 0.001
**Plasma:**										
log Mo	-0.11	**0.27<br>[0.13, 0.40]<br>p < 0.001**	0.10<br>[-0.03, 0.23]<br>p = 0.149	-0.003<br>[-0.01, 0.004]<br>p = 0.445	**0.16<br>[0.008, 0.31]<br>p = 0.039**	0.11<br>[-0.04, 0.26]<br>p = 0.142	-0.006<br>[-0.03, 0.02]<br>p = 0.569	0.10<br>[-0.07, 0.27]<br>p = 0.257	-0.04<br>[-0.18, 0.10]<br>p = 0.575	0.12<br>p < 0.001
Fe	1.48	-0.06<br>[-0.21, 0.08]<br>p = 0.384	-0.07<br>[-0.20, 0.07]<br>p = 0.351	0.001<br>[-0.007, 0.008]<br>p = 0.845	**0.43<br>[0.27, 0.58]<br>p < 0.001**	-0.11<br>[-0.26, 0.05]<br>p = 0.189	-0.005<br>[-0.03, 0.02]<br>p = 0.678	-0.07<br>[-0.25, 0.11]<br>p = 0.429	-0.01<br>[-0.15, 0.13]<br>p = 0.857	0.13<br>p < 0.001
log Zn	6.57	**-0.08<br>[-0.13, -0.04]<br>p < 0.001**	0.007<br>[-0.03, 0.05]<br>p = 0.741	-0.0003<br>[-0.003, 0.002]<br>p = 0.798	0.04<br>[-0.007, 0.09]<br>p = 0.093	-0.04<br>[-0.08, 0.01]<br>p = 0.128	0.004<br>[-0.002, 0.01]<br>p = 0.185	-0.001<br>[-0.06, 0.03]<br>p = 0.969	-0.02<br>[-0.06, 0.03]<br>p = 0.460	0.12<br>p < 0.001
log Cu	6.11	**-0.06<br>[-0.11, -0.003]<br>p = 0.040**	-0.009<br>[-0.06, 0.04]<br>p = 0.747	**0.003<br>[0.0004, 0.006]<br>p = 0.025**	0.02<br>[-0.05, 0.08]<br>p = 0.635	**-0.07<br>[-0.13, -0.01]<br>p = 0.023**	0.008<br>[-0.0002, 0.02]<br>p = 0.057	0.04<br>[-0.03, 0.11]<br>p = 0.228	0.04<br>[-0.02, 0.09]<br>p = 0.193	0.16<br>p < 0.001

log Se	4.60	**-0.13**<br>**[-0.18, -0.08]**<br>**p < 0.001**	-0.001<br>[-0.05, 0.05]<br>p = 0.977	0.002<br>[-0.0004, 0.005]<br>p = 0.093	**0.09**<br>**[0.03, 0.14]**<br>**p = 0.003**	-0.04<br>[-0.10, 0.01]<br>p = 0.116	-0.003<br>[-0.01, 0.005]<br>p = 0.457	-0.01<br>[-0.08, 0.05]<br>p = 0.661	-0.02<br>[-0.07, 0.03]<br>p = 0.533	0.18<br>p < 0.001
log Mg	2.96	-0.02<br>[-0.04, 0.006]<br>p = 0.140	-0.002<br>[-0.02, 0.02]<br>p = 0.843	0.001<br>[-0.00002, 0.002]<br>p = 0.054	0.006<br>[-0.02, 0.03]<br>p = 0.668	-0.01<br>[-0.04, 0.02]<br>p = 0.417	-0.001<br>[-0.005, 0.002]<br>p = 0.512	0.02<br>[-0.01, 0.05]<br>p = 0.201	-0.003<br>[-0.03, 0.02]<br>p = 0.825	0.02<br>p = 0.226
log Ca	4.49	-0.01<br>[-0.02, 0.001]<br>p = 0.078	**0.01**<br>**[0.003, 0.03]**<br>**p = 0.015**	-0.0003<br>[-0.001, 0.0003]<br>p = 0.300	**0.03**<br>**[0.01, 0.04]**<br>**p < 0.001**	-0.01<br>[-0.02, 0.005]<br>p = 0.249	0.001<br>[-0.001, 0.003]<br>p = 0.346	-0.004<br>[-0.02, 0.01]<br>p = 0.595	0.005<br>[-0.007, 0.02]<br>p = 0.404	0.16<br>p < 0.001
log I	3.57	**-0.09**<br>**[-0.17, -0.01]**<br>**p = 0.019**	-0.06<br>[-0.14, 0.01]<br>p = 0.089	0.001<br>[-0.003, 0.005<br>p = 0.740	-0.02<br>[-0.11, 0.06]<br>p = 0.616	-0.04<br>[-0.12, 0.05]<br>p = 0.382	0.008<br>[-0.004, 0.02]<br>p = 0.184	-0.006<br>[-0.10, 0.09]<br>p = 0.901	-0.02<br>[-0.09, 0.06]<br>p = 0.660	0.04<br>p = 0.086
log Al†	1.36	0.02<br>[-0.16, 0.20]<br>p = 0.835	0.01<br>[-0.14, 0.17]<br>p = 0.868	-0.003<br>[-0.009, 0.004]<br>p = 0.388	0.13<br>[-0.007, 0.26]<br>p = 0.062	-0.04<br>[-0.19, 0.10]<br>p = 0.554	-0.007<br>[-0.03, 0.01]<br>p = 0.491	-0.005<br>[-0.15, 0.14]<br>p = 0.948	**-0.13**<br>**[-0.25, - 0.001]**<br>**p = 0.048**	
log Li	-1.87	-0.06<br>[-0.44, 0.32]<br>p = 0.752	-0.28<br>[-0.64, 0.09]<br>p = 0.137	0.02<br>[-0.0004, 0.04]<br>p = 0.054	0.36<br>[-0.06, 0.78]<br>p = 0.093	0.11<br>[-0.30, 0.52]<br>p = 0.605	0.05<br>[-0.006, 0.11]<br>p = 0.080	-0.06<br>[-0.53, 0.42]<br>p = 0.809	-0.34<br>[-0.72, 0.03]<br>p = 0.071	0.06<br>p = 0.028
log Sr	2.78	**0.21**<br>**[0.11, 0.31]**<br>**p < 0.001**	0.001<br>[-0.09, 0.09]<br>p = 0.989	**0.006**<br>**[0.001, 0.01]**<br>**p = 0.015**	0.05<br>[-0.06, 0.15]<br>p = 0.400	-0.02<br>[-0.13, 0.09]<br>p = 0.707	-0.007<br>[-0.02, 0.008]<br>p = 0.357	0.11<br>[-0.02, 0.23]<br>p = 0.085	0.06<br>[-0.04, 0.15]<br>p = 0.237	0.14<br>p < 0.001
log Sn	-1.27	**-0.23**<br>**[-0.35, -0.10]**<br>**p < 0.001**	-0.09<br>[-0.21, 0.03]<br>p = 0.137	0.006<br>[-0.0002, 0.01]<br>p = 0.056	**0.15**<br>**[0.02, 0.29]**<br>**p = 0.027**	0.06<br>[-0.07, 0.19]<br>p = 0.364	0.008<br>[-0.01, 0.03]<br>p = 0.426	-0.003<br>[-0.16, 0.15]<br>p = 0.969	**-0.14**<br>**[-0.26, -0.02]**<br>**p = 0.021**	0.16<br>p < 0.001
log Ba	-0.85	0.02<br>[-0.14, 0.17]<br>p = 0.803	-0.03<br>[-0.18, 0.12]<br>p = 0.666	0.006<br>[-0.002, 0.01]<br>p = 0.141	-0.03<br>[-0.20, 0.14]<br>p = 0.714	-0.05<br>[-0.22, 0.12]<br>p = 0.580	-0.005<br>[-0.03, 0.02]<br>p = 0.663	0.09<br>[-0.11, 0.28]<br>p = 0.380	0.11<br>[-0.05, 0.26]<br>p = 0.172	0<br>p = 0.506

Each row corresponds to a separate linear regression model. For each model, regression coefficient with 95% confidence interval and p-value is shown for all independent variables included in the model. Significant associations ($p < 0.05$) are shown in bold font.

* Variables were categorized as follows: dominant (CC) genotype (referent category) *vs*. T allele carriers (CT+TT) for genotype; primary or secondary school (referent category) *vs*. university degree or higher.

§ Adjusted R^2 and model p-value were not available from the R package *lodi* used for censored likelihood multiple imputation.

† Model for plasma Al additionally included the interaction term between the type of diet and genotype with an estimated coefficient -0.34 [-0.62, -0.06] (p = 0.019).

Table S6 Results of multiple regression analysis for cholesterol, folate, glucose, triglycerides, and B12 in plasma

	**Intercept**	**Vegetarian diet**	**Genotype***	**Age**	**Male sex**	**Smoking**	**Body mass index**	**Education level***	**Supplements intake†**	**Adjusted R^2; model p-value**
log total cholesterol	0.98	**-0.11<br>[-0.17, -0.04]<br>p = 0.001**	0.03<br>[-0.03, 0.09]<br>p = 0.389	**0.01<br>[0.003, 0.009]<br>p = 0.001**	-0.02<br>[-0.09, 0.04]<br>p = 0.480	-0.05<br>[-0.12, 0.01]<br>p = 0.124	**0.01<br>[0.004, 0.02]<br>p = 0.006**	0.02<br>[-0.05, 0.10]<br>p = 0.535		0.25<br>p < 0.001
log HDL cholesterol§	0.60	**-0.14<br>[-0.25, -0.01]<br>p = 0.028**	-0.07<br>[-0.17, 0.03]<br>p = 0.178	0.003<br>[-0.001, 0.008]<br>p = 0.142	-0.07<br>[-0.16, 0.02]<br>p = 0.128	-0.04<br>[-0.13, 0.05]<br>p = 0.357	-0.01<br>[-0.02, 0.002]<br>p = 0.117	-0.03<br>[-0.14, 0.07]<br>p = 0.534		0.03<br>p = 0.104
log LDL cholesterol	-0.44	**-0.23<br>[-0.33, -0.13]<br>p < 0.001**	0.01<br>[-0.09, 0.11]<br>p = 0.834	**0.009<br>[0.004, 0.01]<br>p = 0.001**	0.06<br>[-0.05, 0.17]<br>p = 0.290	-0.07<br>[-0.18, 0.04]<br>p = 0.202	**0.03<br>[0.01, 0.04]<br>p < 0.001**	0.04<br>[-0.09, 0.16]<br>p = 0.588		0.32<br>p < 0.001
log glucose	1.33	-0.02<br>[-0.05, 0.006]<br>p = 0.127	0.008<br>[-0.02, 0.04]<br>p = 0.572	0.001<br>[-0.001, 0.002]<br>p = 0.471	0.02<br>[-0.01, 0.05]<br>p = 0.181	-0.01<br>[-0.04, 0.02]<br>p = 0.414	**0.009<br>[0.005, 0.01]<br>p < 0.001**	-0.03<br>[-0.07, 0.006]<br>p = 0.097		0.17<br>p < 0.001
log triglycerides	-1.01	0.09<br>[-0.03, 0.20]<br>p = 0.153	-0.02<br>[-0.13, 0.10]<br>p = 0.774	**0.006<br>[0.0002, 0.01]<br>p = 0.043**	0.08<br>[-0.04, 0.21]<br>p = 0.201	**0.18<br>[0.05, 0.31]<br>p = 0.007**	**0.03<br>[0.009, 0.04]<br>p = 0.003**	-0.06<br>[-0.21, 0.09]<br>p = 0.426		0.12<br>p < 0.001
log folate	2.37	0.13<br>[-0.01, 0.26]<br>p = 0.073	**-0.19<br>[-0.32, -0.06]<br>p = 0.005**	0.003<br>[-0.005, 0.01]<br>p = 0.500	0.03<br>[-0.12, 0.18]<br>p = 0.708	0.002<br>[-0.15, 0.15]<br>p = 0.981	0.003<br>[-0.02, 0.02]<br>p = 0.791	0.14<br>[-0.03, 0.32]<br>p = 0.108	0.12<br>[-0.11, 0.35]<br>p = 0.313	0.05<br>p = 0.045
log vitamin B_{12}	5.76	**-0.26<br>[-0.38, -0.13]<br>p < 0.001**	0.04<br>[-0.07, 0.14]<br>p = 0.514	-0.003<br>[-0.009, 0.003]<br>p = 0.339	0.02<br>[-0.11, 0.15]<br>p = 0.743	-0.05<br>[-0.18, 0.07]<br>p = 0.406	0.008<br>[-0.01, 0.02]<br>p = 0.382	-0.02<br>[-0.17, 0.12]<br>p = 0.849	**0.22<br>[0.10, 0.34]<br>p = 0.001**	0.10<br>p = 0.002

Each row corresponds to a separate linear regression model. For each model, regression coefficient with 95% confidence interval and p-value is shown for all independent variables included in the model. Significant associations ($p < 0.05$) are shown in bold font.

*Variables were categorized as follows: dominant (CC) genotype (referent category) *vs*. T allele carriers (CT+TT) for genotype; primary or secondary school (referent category) *vs*. university degree or higher.

† Intake of folic acid as an independent variable was included in the regression model for folate, while intake of vitamin B_{12} was included in the regression model for vitamin B_{12}.

§ Model for HDL additionally included the interaction term between the type of diet and genotype, with an estimated coefficient 0.16 [0.003, 0.33] (p = 0.046).

Table S7 Results of multiple regression analysis for amino acids in plasma

	Intercept	Vegetarian diet	Genotype*	Age	Male sex	Smoking	Body mass index	Education level*	Supplements intake	Adjusted R^2; model p-value§
log alanine†	5.37	0.05<br>[-0.07, 0.18]<br>p = 0.3801	-0.10<br>[-0.20, 0.008]<br>p = 0.069	0.001<br>[-0.004, 0.005]<br>p = 0.782	0.02<br>[-0.08, 0.11]<br>p = 0.697	0.05<br>[-0.04, 0.14]<br>p = 0.276	**0.02<br>[0.008, 0.03]<br>p = 0.001**	0.03<br>[-0.08, 0.13]<br>p = 0.643	-0.05<br>[-0.17, 0.08]<br>p = 0.446	0.11<br>p = 0.002
log sarcosine	1.65	**-0.53<br>[-0.71, -0.34]<br>p < 0.001**	-0.13<br>[-0.30, 0.04]<br>p = 0.137	-0.003<br>[-0.01, 0.006]<br>p = 0.474	-0.008<br>[-0.21, 0.19]<br>p = 0.939	-0.03<br>[-0.22, 0.16]<br>p = 0.734	**0.03<br>[0.006, 0.06]<br>p = 0.015**	0.06<br>[-0.16, 0.28]<br>p = 0.578	0.14<br>[-0.12, 0.39]<br>p = 0.293	0.24<br>p < 0.001
log glycine†	5.66	0.03<br>[-0.09, 0.14]<br>p = 0.664	-0.01<br>[-0.11, 0.09]<br>p = 0.797	0.001<br>[-0.003, 0.005]<br>p = 0.602	-0.06<br>[-0.15, 0.03]<br>p = 0.210	0.04<br>[-0.05, 0.12]<br>p = 0.404	-0.006<br>[-0.02, 0.005]<br>p = 0.298	-0.07<br>[-0.17, 0.03]<br>p = 0.163	0.02<br>[-0.10, 0.14]<br>p = 0.737	0.09<br>p = 0.007
log *α*-aminobutyric acid	3.22	**-0.29<br>[-0.43, -0.16]<br>p < 0.001**	-0.06<br>[-0.19, 0.06]<br>p = 0.321	-0.003<br>[-0.009, 0.004]<br>p = 0.460	0.06<br>[-0.09, 0.21]<br>p = 0.453	0.02<br>[-0.12, 0.16]<br>p = 0.453	**0.02<br>[0.004, 0.04]<br>p = 0.019**	**0.17<br>[0.007, 0.34]<br>p = 0.041**	-0.05<br>[-0.25, 0.14]<br>p = 0.581	0.19<br>p < 0.001
log valine	5.82	0.007<br>[-0.06, 0.08]<br>p = 0.839	0.007<br>[-0.06, 0.07]<br>p = 0.826	-0.001<br>[-0.005, 0.002]<br>p = 0.528	**0.17<br>[0.09, 0.25]<br>p < 0.001**	0.02<br>[-0.05, 0.10]<br>p = 0.579	**0.02<br>[0.005, 0.03]<br>p = 0.003**	0.05<br>[-0.04, 0.13]<br>p = 0.276	-0.09<br>[-0.19, 0.01]<br>p = 0.081	0.17<br>p < 0.001
β-aminoisobutyric acid	2.13	-0.08<br>[-0.82, 0.66]<br>p = 0.836	-0.05<br>[-0.74, 0.64]<br>p = 0.877	0.01<br>[-0.03, 0.05]<br>p = 0.531	**1.10<br>[0.27, 1.91]<br>p = 0.009**	0.02<br>[-0.77, 0.80]<br>p = 0.965	0.05<br>[-0.06, 0.15]<br>p = 0.403	-0.02<br>[-0.92, 0.89]<br>p = 0.968	-0.15<br>[-1.21, 0.91]<br>p = 0.781	0.02<br>p = 0.233
leucine	84.2	-6.53<br>[-14.5, 1.48]<br>p = 0.109	5.21<br>[-1.23, 13.6]<br>p = 0.101	**-0.47<br>[-0.88, -0.06]<br>p = 0.026**	**24.5<br>[15.7, 33.3]<br>p < 0.001**	6.03<br>[-2.43, 14.5]<br>p = 0.161	1.14<br>[-0.03, 2.31]<br>p = 0.056	2.83<br>[-6.93, 12.6]<br>p = 0.568	-4.83<br>[-16.2, 6.56]<br>p = 0.4040	0.25<br>p < 0.001
log allo-*iso*-leucine	-0.30	**0.16<br>[0.03, 0.29]<br>p = 0.014**	-0.02<br>[-0.14, 0.10]<br>p = 0.750	-0.001<br>[-0.007, 0.006]<br>p = 0.864	**0.18<br>[0.04, 0.32]<br>p = 0.010**	-0.05<br>[-0.18, 0.08]<br>p = 0.458	0.02<br>[-0.004, 0.03]<br>p = 0.123	0.08<br>[-0.07, 0.24]<br>p = 0.304	-0.09<br>[-0.27, 0.09]<br>p = 0.314	0.05<br>p = 0.042
log *iso*-leucine	3.83	0.06<br>[-0.03, 0.14]<br>p = 0.198	0.06<br>[-0.02, 0.14]<br>p = 0.130	-0.004<br>[-0.008, 0.0004]<br>p = 0.072	**0.21<br>[0.12, 0.30]<br>p < 0.001**	0.07<br>[-0.01, 0.16]<br>p = 0.100	0.01<br>[-0.002, 0.02]<br>p = 0.107	0.01<br>[-0.09, 0.11]<br>p = 0.790	-0.03<br>[-0.15, 0.09]<br>p = 0.643	0.18<br>p < 0.001
log threonine	4.28	0.08<br>[-0.11, 0.27]<br>p = 0.397	-0.05<br>[-0.23, 0.16]<br>p = 0.559	0.002<br>[-0.008, 0.01]<br>p = 0.676	0.10<br>[-0.11, 0.31]<br>p = 0.364	0.10<br>[-0.10, 0.31]<br>p = 0.313	0.005<br>[-0.02, 0.03]<br>p = 0.702	-0.05<br>[-0.28, 0.18]<br>p = 0.674	0.04<br>[-0.23, 0.31]<br>p = 0.776	0<br>p = 0.840
log serine	4.68	0.03<br>[-0.009, 0.16]<br>p = 0.578	0.001<br>[-0.11, 0.11]<br>p = 0.985	-0.0003<br>[-0.006, 0.006]<br>p = 0.921	-0.07<br>[-0.20, 0.06]<br>p = 0.295	**0.14<br>[0.01, 0.27]<br>p = 0.031**	-0.004<br>[-0.02, 0.01]<br>p = 0.649	-0.05<br>[-0.19, 0.10]<br>p = 0.509	0.04<br>[-0.14, 0.21]<br>p = 0.686	0<br>p = 0.465
log proline	4.99	**0.26<br>[0.15, 0.36]<br>p < 0.001**	**0.11<br>[0.009, 0.21]<br>p = 0.032**	-0.003<br>[-0.009, 0.002]<br>p = 0.232	**0.15<br>[0.03, 0.27]<br>p = 0.013**	**0.13<br>[0.02, 0.25]<br>p = 0.020**	0.02<br>[-0.001, 0.03]<br>p = 0.066	-0.07<br>[-0.20, 0.06]<br>p = 0.305	-0.07<br>[-0.22, 0.08]<br>p = 0.364	0.21<br>p < 0.001
log asparagine	4.16	**0.15<br>[0.06, 0.25]<br>p = 0.001**	-0.02<br>[-0.11, 0.06]<br>p = 0.590	0.001<br>[-0.004, 0.005]<br>p = 0.752	0.07<br>[-0.03, 0.17]<br>p = 0.184	0.04<br>[-0.06, 0.13]<br>p = 0.453	**-0.02<br>[-0.03, -0.001]<br>p = 0.034**	-0.07<br>[-0.18, 0.04]<br>p = 0.207	0.04<br>[-0.09, 0.17]<br>p = 0.580	0.12<br>p < 0.001

log aspartic acid	1.54	0.14<br>[-0.06, 0.34]<br>p = 0.147	**0.20**<br>**[0.02, 0.38]**<br>**p = 0.027**	-0.001<br>[-0.01, 0.009]<br>p = 0.859	0.03<br>[-0.19, 0.24]<br>p = 0.802	**0.25**<br>**[0.04, 0.45]**<br>**p = 0.018**	0.004<br>[-0.02, 0.03]<br>p = 0.786	-0.004<br>[-0.24, 0.23]<br>p = 0.971	-0.07<br>[-0.35, 0.20]<br>p = 0.610	0.03<br>p = 0.131
log methionine	3.46	0.02<br>[-0.04, 0.08]<br>p = 0.452	0.007<br>[-0.04, 0.06]<br>p = 0.794	-0.001<br>[-0.004, 0.002]<br>p = 0.588	**0.08**<br>**[0.02, 0.14]**<br>**p = 0.012**	0.05<br>[-0.01, 0.11]<br>p = 0.126	-0.003<br>[-0.01, 0.006]<br>p = 0.511	-0.02<br>[-0.08, 0.05]<br>p = 0.666	**-0.09**<br>**[-0.17, -0.005]**<br>**p = 0.037**	0.04<br>p = 0.080
4-hydroxyproline	20.7	**-1.58**<br>**[-3.09, -0.06]**<br>**p = 0.042**	1.18<br>[-0.23, 2.59]<br>p = 0.100	-0.04<br>[-0.11, 0.04]<br>p = 0.336	-0.46<br>[-2. 12, 1.21]<br>p = 0.587	1.22<br>[-0.38, 2.82]<br>p = 0.134	0.04<br>[-0.19, 0.26]<br>p = 0.757	-0.82<br>[-2.67, 1.03]<br>p = 0.382	0.74<br>[-1.42, 2.89]<br>p = 0.500	0.02<br>p = 0.206
log phenylalanine	4.08	0.05<br>[-0.06, 0.16]<br>p = 0.371	0.04<br>[-0.06, 0.14]<br>p = 0.471	-0.005<br>[-0.01, 0.0004]<br>p = 0.072	**0.17**<br>**[0.05, 0.28]**<br>**p = 0.006**	0.05<br>[-0.06, 0.16]<br>p = 0.409	0.001<br>[-0.01, 0.02]<br>p = 0.937	0.11<br>[-0.02, 0.24]<br>p = 0.109	-0.09<br>[-0.24, 0.06]<br>p = 0.249	0.05<br>p = 0.047
log glutamic acid	3.38	**0.19**<br>**[0.09, 0.29]**<br>**p < 0.001**	**0.11**<br>**[0.02, 0.20]**<br>**p = 0.020**	**0.006**<br>**[0.0004, 0.01]**<br>**p = 0.036**	**0.18**<br>**[0.07, 0.29]**<br>**p = 0.001**	**0.17**<br>**[0.07, 0.28]**<br>**p = 0.002**	0.008<br>[-0.006, 0.02]<br>p = 0.256	-0.04<br>[-0.16, 0.08]<br>p = 0.501	-0.04<br>[-0.18, 0.10]<br>p = 0.569	0.22<br>p < 0.001
α-aminoadipic acid	1.48	-0.50<br>[-1.32, 0.32]<br>p = 0.228	-0.003<br>[-0.76, 0.75]<br>p = 0.994	-0.008<br>[-0.05, 0.03]<br>p = 0.688	**1.04**<br>**[0.13, 1.95]**<br>**p = 0.025**	**1.10**<br>**[0.23, 1.97]**<br>**p = 0.014**	0.05<br>[-0.07, 0.17]<br>p = 0.423	0.43<br>[-0.56, 1.42]<br>p = 0.394	0.09<br>[-1.08, 1.26]<br>p = 0.874	
cysteine	-12.7	6.00<br>[-10.9, 22.9]<br>p = 0.483	3.78<br>[-13.4, 20.9]<br>p = 0.660	-0.52<br>[-1.42, 0.37]<br>p = 0.248	-17.8<br>[-42.6, 7.0]<br>p = 0.148	11.6<br>[-7.2, 30.4]<br>p = 0.222	1.43<br>[-1.10, 3.97]<br>p = 0.264	9. 78<br>[-11.5, 31.1]<br>p = 0.364	7.09<br>[-16.3, 30.5]<br>p = 0.550	
log ornithine	4.05	**0.11**<br>**[0.01, 0.22]**<br>**p = 0.030**	-0.03<br>[-0.12, 0.07]<br>p = 0.591	**0.005**<br>**[0.0001, 0.01]**<br>**p = 0.046**	**0.12**<br>**[0.01, 0.24]**<br>**p = 0.029**	**0.11**<br>**[0.004, 0.22]**<br>**p = 0.042**	0.003<br>[-0.01, 0.02]<br>p = 0.656	0.02<br>[-0.11, 0.14]<br>p = 0.793	0.03<br>[-0.12, 0.17]<br>p = 0.690	0.07<br>p = 0.013
log lysine	4.76	**-0.12**<br>**[-0.21, -0.03]**<br>**p = 0.008**	-0.05<br>[-0.13, 0.03]<br>p = 0.197	0.004<br>[-0.0008, 0.008]<br>p = 0.112	**0.12**<br>**[0.03, 0.22]**<br>**p = 0.011**	0.02<br>[-0.07, 0.11]<br>p = 0.648	0.007<br>[-0.006, 0.02]<br>p = 0.308	0.05<br>[-0.05, 0.16]<br>p = 0.344	0.01<br>[-0.11, 0.13]<br>p = 0.875	0.12<br>p < 0.001
log histidine	4.09	0.07<br>[-0.03, 0.17]<br>p = 0.164	-0.09<br>[-0.18, 0.008]<br>p = 0.073	0.001<br>[-0.004, 0.006]<br>p = 0.810	0.09<br>[-0.02, 0.20]<br>p = 0.131	0.02<br>[-0.09, 0.12]<br>p = 0.780	0.003<br>[-0.01, 0.02]<br>p = 0.689	0.05<br>[-0.07, 0.18]<br>p = 0.408	0.02<br>[-0.13, 0.16]<br>p = 0.807	0.01<br>p = 0.320
log tyrosine	4.02	0.08<br>[-0.04, 0.20]<br>p = 0.175	-0.07<br>[-0.18, 0.04]<br>p = 0.220	0.0003<br>[-0.006, 0.006]<br>p = 0.929	**0.18**<br>**[0.05, 0.31]**<br>**p = 0.008**	0.004<br>[-0.12, 0.13]<br>p = 0.946	0.01<br>[-0.004, 0.03]<br>p = 0.155	0.02<br>[-0.13, 0.16]<br>p = 0.840	-0.06<br>[-0.23, 0.11]<br>p = 0.469	0.04<br>p = 0.095
log tryptophan	3.88	0.02<br>[-0.09, 0.13]<br>p = 0.711	-0.03<br>[-0.14, 0.07]<br>p = 0.545	-0.004<br>[-0.01, 0.001]<br>p = 0.139	**0.14**<br>**[0.02, 0.26]**<br>**p = 0.022**	-0.02<br>[-0.13, 0.10]<br>p = 0.798	0.008<br>[-0.008, 0.02]<br>p = 0.316	0.06<br>[-0.08, 0.19]<br>p = 0.409	-0.02<br>[-0.18, 0.14]<br>p = 0.792	0.02<br>p = 0.232

Each row corresponds to a separate linear regression model. For each model, regression coefficient with 95% confidence interval and p-value is shown for all independent variables included in the model. Significant associations ($p < 0.05$) are shown in bold font.

* Variables were categorized as follows: dominant (CC) genotype (referent category) *vs*. T allele carriers (CT+TT) for genotype; primary or secondary school (referent category) *vs*. university degree or higher.

§ Adjusted R^2 and model p-value were not available from the R package *lodi* used for censored likelihood multiple imputation.

† Model for alanine and glycine additionally included the interaction term between the type of diet and genotype with an estimated coefficient 0.21 [0.04, 0.37] (p = 0.013), 0.16 [0.005, 0.32] (p = 0.043), respectively.

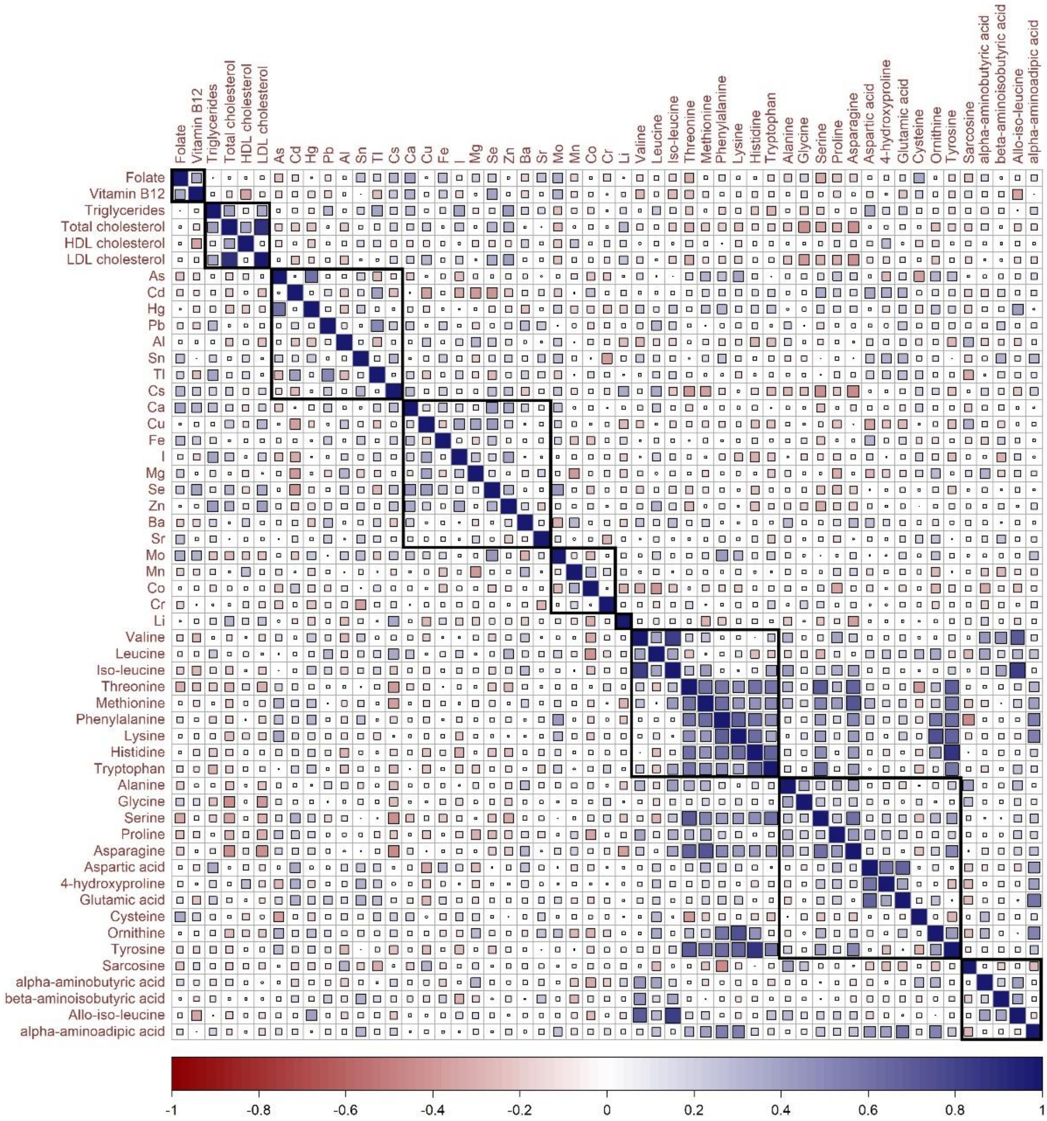

Fig. S2. Spearman's correlation coefficients between measured parameters in non-vegetarian individuals with the CC genotype. Parameters were grouped according to their nutritional characteristics and/or biological functions as follows: B vitamins (folates, vitamin B12); lipid profile (triglycerides, total cholesterol, HDL and LDL cholesterol); toxic elements (As, Cd, Hg, Pb, Al, Sn, Tl, Cs); essential elements (Ca, Cu, Fe, I, Mg, Se, Zn, Ba, Sr); trace essential elements (Mo, Mn, Co, Cr); essential amino acids (valine, leucine, iso-leucine, threonine, methionine, phenylalanine, lysine, histidine, tryptophan); non-essential amino acids (alanine, glycine, serine, proline, asparagine, aspartic acid, 4-hydroxyproline, glutamic acid, cysteine, ornithine, tyrosine); and non-proteinogenic amino acids (sarcosine, alpha-aminobutyric acid, beta-aminoisobutyric acid, allo-iso-leucine, alpha-aminoadipic acid). Dark blue indicates positive correlations and red indicates negative correlations, with the intensity of the color reflecting strength of the association.

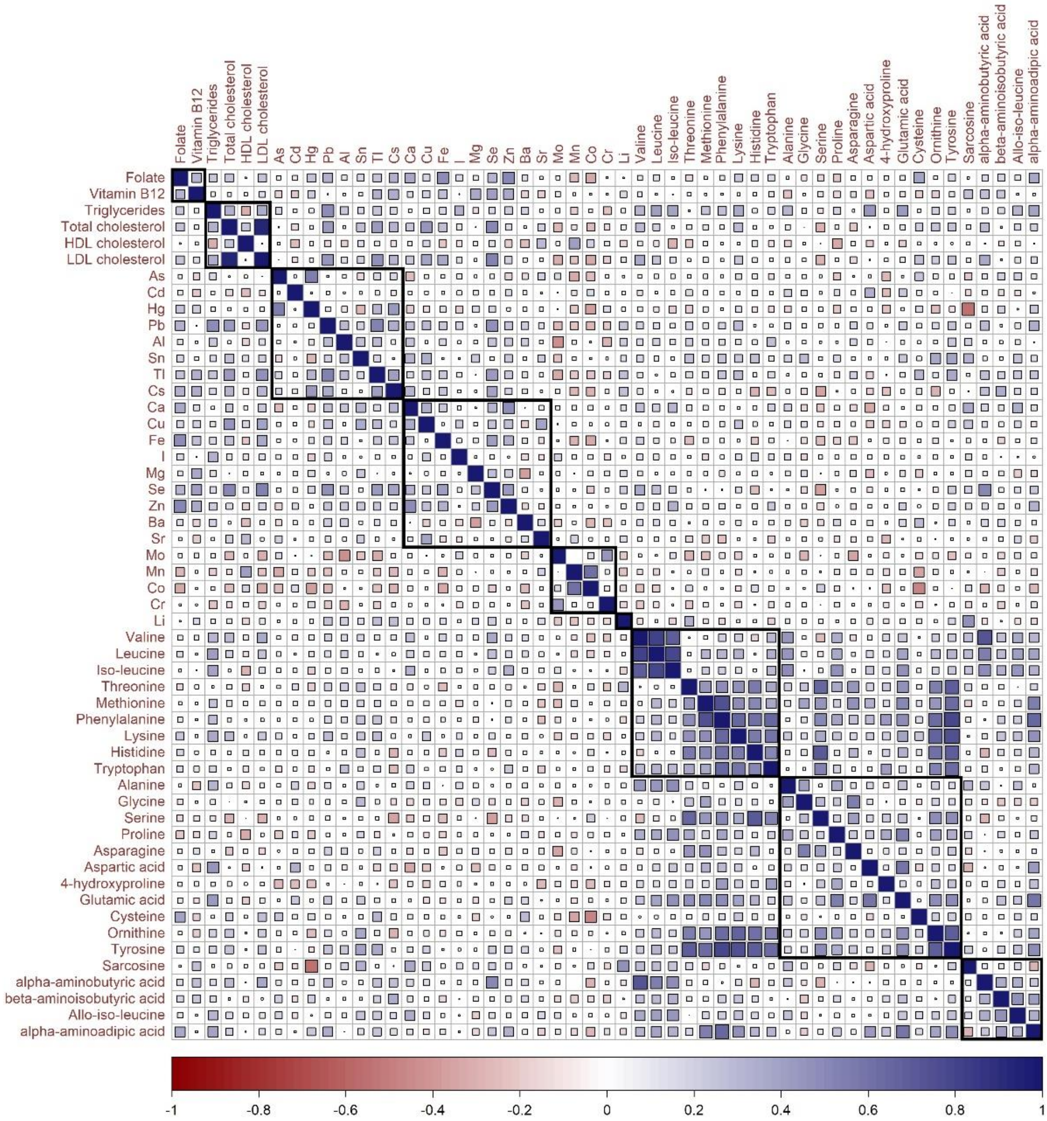

Fig. S3. Spearman's correlation coefficients between measured parameters in non-vegetarian individuals with the CT+TT genotype. Parameters were grouped according to their nutritional characteristics and/or biological functions as follows: B vitamins (folates, vitamin B12); lipid profile (triglycerides, total cholesterol, HDL and LDL cholesterol); toxic elements (As, Cd, Hg, Pb, Al, Sn, Tl, Cs); essential elements (Ca, Cu, Fe, I, Mg, Se, Zn, Ba, Sr); trace essential elements (Mo, Mn, Co, Cr); essential amino acids (valine, leucine, iso-leucine, threonine, methionine, phenylalanine, lysine, histidine, tryptophan); non-essential amino acids (alanine, glycine, serine, proline, asparagine, aspartic acid, 4-hydroxyproline, glutamic acid, cysteine, ornithine, tyrosine); and non-proteinogenic amino acids (sarcosine, alpha-aminobutyric acid, beta-aminoisobutyric acid, allo-iso-leucine, alpha-aminoadipic acid). Dark blue indicates positive correlations and red indicates negative correlations, with the intensity of the color reflecting strength of the association.

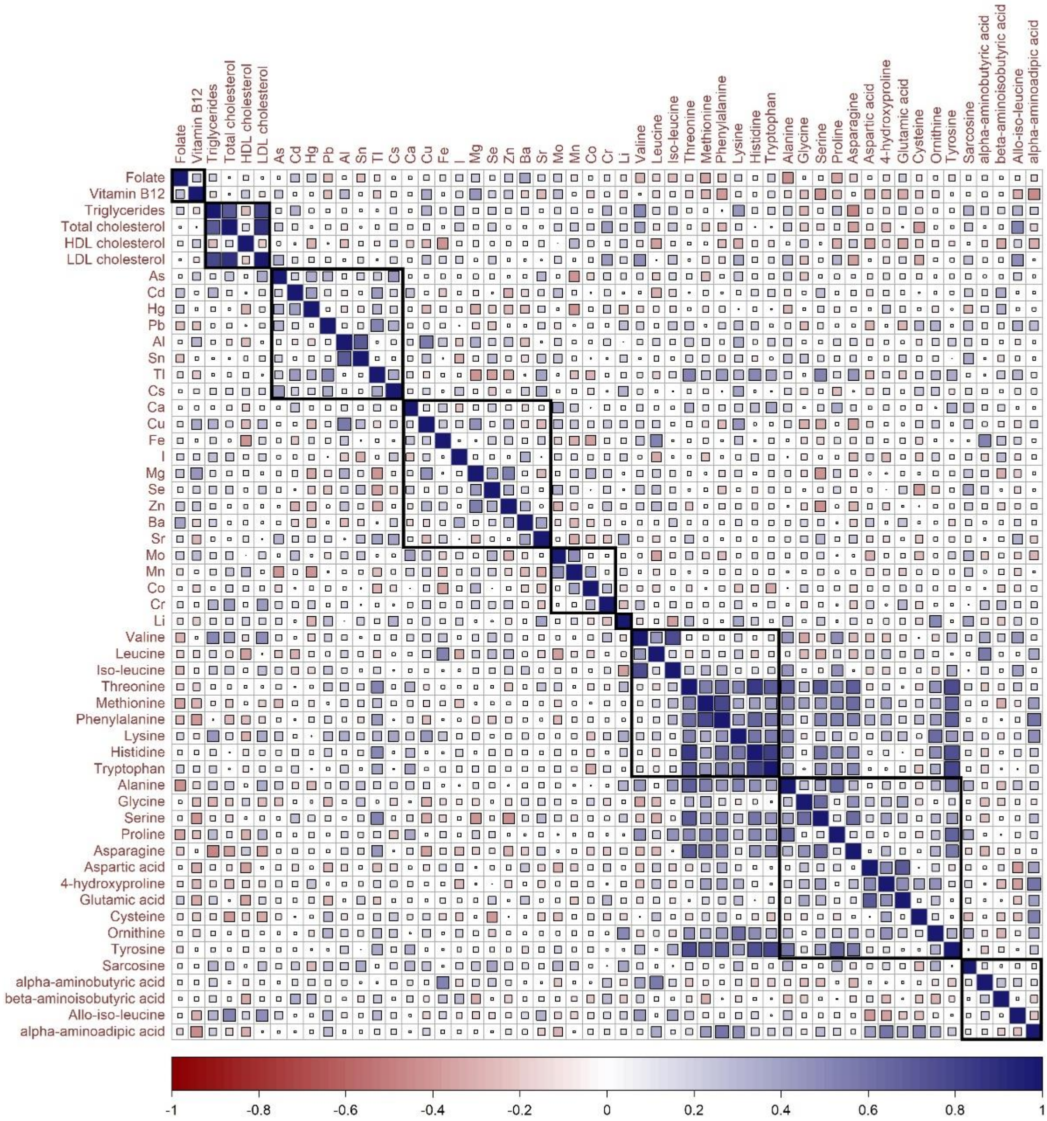

Fig. S4. Spearman's correlation coefficients between measured parameters in the vegetarian individuals with the CC genotype. Parameters were grouped according to their nutritional characteristics and/or biological function as follows: B vitamins (folates, vitamin B12); lipid profile (triglycerides, total cholesterol, HDL and LDL cholesterol); toxic elements (As, Cd, Hg, Pb, Al, Sn, Tl, Cs); essential elements (Ca, Cu, Fe, I, Mg, Se, Zn, Ba, Sr); trace essential elements (Mo, Mn, Co, Cr); essential amino acids (valine, leucine, iso-leucine, threonine, methionine, phenylalanine, lysine, histidine, tryptophan); non-essential amino acids (alanine, glycine, serine, proline, asparagine, aspartic acid, 4-hydroxyproline, glutamic acid, cysteine, ornithine, tyrosine); and non-proteinogenic amino acids (sarcosine, alpha-aminobutyric acid, beta-aminoisobutyric acid, allo-iso-leucine, alpha-aminoadipic acid). Dark blue indicates positive correlations and red indicates negative correlations, with the intensity of the color reflecting strength of the association.

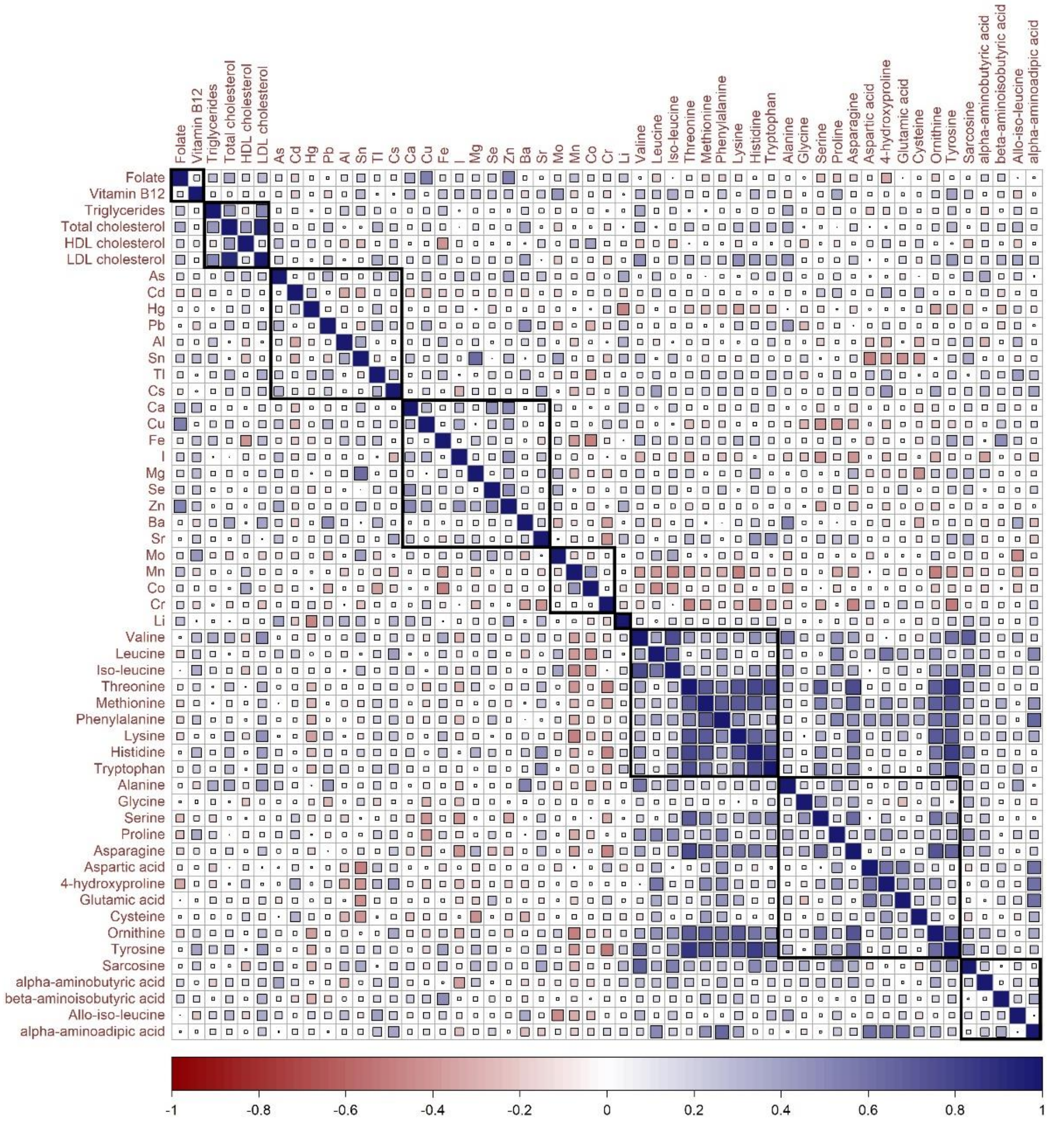

Fig. S5. Spearman's correlation coefficients between measured parameters in the vegetarian individuals with the CT+TT genotype. Parameters were grouped according to their nutritional characteristics and/or biological function as follows: B vitamins (folates, vitamin B12); lipid profile (triglycerides, total cholesterol, HDL and LDL cholesterol); toxic elements (As, Cd, Hg, Pb, Al, Sn, Tl, Cs); essential elements (Ca, Cu, Fe, I, Mg, Se, Zn, Ba, Sr); trace essential elements (Mo, Mn, Co, Cr); essential amino acids (valine, leucine, iso-leucine, threonine, methionine, phenylalanine, lysine, histidine, tryptophan); non-essential amino acids (alanine, glycine, serine, proline, asparagine, aspartic acid, 4-hydroxyproline, glutamic acid, cysteine, ornithine, tyrosine); and non-proteinogenic amino acids (sarcosine, alpha-aminobutyric acid, beta-aminoisobutyric acid, allo-iso-leucine, alpha-aminoadipic acid). Dark blue indicates positive correlations and red indicates negative correlations, with the intensity of the color reflecting strength of the association
